# Growth and Hormonal Responses to Salicylic Acid and Calcium Chloride Seed Priming in Domestic and Wild Salt-Tolerant Barley Species Under Saline Conditions

**DOI:** 10.3390/plants15010064

**Published:** 2025-12-25

**Authors:** Rim Ben Youssef, Nahida Jelali, Purificación Andrea Martínez-Melgarejo, Alfonso Albacete, Chedly Abdelly, Francisco Pérez-Alfocea, Cristina Martínez-Andújar

**Affiliations:** 1 Laboratory of Extremophile Plants, Centre of Biotechnology of Borj-Cédria (CBBC), P.O. Box 901, Hammam-Lif 2050, Tunisia; rim.benyoussef@hotmail.com (R.B.Y.); nahidajelali@gmail.com (N.J.); abdelly.chedly@gmail.com (C.A.); 2 Faculty of Sciences of Tunis, University of Tunis El Manar, Tunis 1060, Tunisia; 3 Group of Plant Hormone, CEBAS-CSIC, 30100 Murcia, Spain; pmelgarejo@cebas.csic.es (P.A.M.-M.); alfocea@cebas.csic.es (F.P.-A.); 4 Institute for Agroenvironmental Research and Development of Murcia (IMIDA), c/Mayor s/n, 30150 Murcia, Spain; alfonsoa.albacete@carm.es

**Keywords:** plant hormone, salinity stress, seed priming, growth recovery, *Hordeum vulgare*, *Hordeum maritimum*

## Abstract

Salinity is among the main abiotic constraints limiting crop productivity worldwide. Salt tolerance can be improved by introducing adaptive traits from wild species and enhancing pre-existing salt-adaptive mechanisms through priming. This study evaluated the beneficial effect of salicylic acid (SA, 1.25 mM) and calcium chloride (CaCl_2_, 5 mM) seed priming on plant growth under salinity in the domestic barley *Hordeum vulgare* (*Hv*) and the wild, salt-adapted *Hordeum maritimum* (*Hm*). Primed plants were grown under control, 100 and 200 mM sodium chloride (NaCl) for two weeks. Growth and hormone profiling were performed. *Hv* showed higher growth inhibition than *Hm* but was more responsive to stress alleviation by priming, particularly with SA, which increased biomass by up to 47% at 200 mM NaCl. The contrasting responses of both species reflected distinct hormonal strategies. The intrinsic salt tolerance of *Hm* appears linked to high constitutive levels of stress- and growth-related hormones. In *Hv*, growth recovery under salinity following priming was associated with hormonal reprogramming, involving reduced abscisic acid (ABA) accumulation and enhanced levels of growth-promoting hormones (indole-3-acetic acid (IAA), trans-zeatin (tZ), and isopentenyl adenine (iP)), especially in roots. Hormonal changes mediated by priming are analyzed in relation to adaptive growth responses and species’ ecological origins.

## 1. Introduction

In general, plant species are exposed to a wide range of biotic and abiotic constraints that adversely affect growth and productivity. In calcareous saline soils in semi-arid and arid regions worldwide, salt stress represents one of the most serious challenges to agriculture [1], as it induces morphological, physiological, biochemical and molecular alterations in plants [1]. Improving the use of such marginal resources therefore requires a better understanding of the limiting effects of salinity on plant development. Plant growth is initially constrained by a reduction in soil water potential (osmotic phase), and is subsequently further impaired by ion toxicity resulting from the accumulation of sodium and chloride ions in leaves (ionic phase) [2]. In addition, salinity disrupts the uptake of essential nutrients (e.g., NO_3_^−^, K^+^, Ca^2+^, and Mg^2+^), thereby disturbing plant mineral nutrition through competition with sodium and chloride ions [3].

Furthermore, salt stress has been widely shown to affect plant growth by altering hormonal homeostasis of plants [4,5,6] and given the central role of hormonal regulation in determining plant performance under salinity, considerable attention has been devoted to understanding these mechanisms in various crops and model species [7]. Under non-stress conditions, plant growth is regulated by a coordinated balance between growth-promoting hormones (auxins, cytokinins, CKs, and gibberellins, GAs) and stress-related hormones (abscisic acid, ABA; jasmonic acid, JA; and ethylene). Salinity disrupts this equilibrium, leading to increased accumulation of ABA and ethylene precursors and a concomitant reduction the synthesis and transport of growth-promoting hormones (auxins and cytokinins) [4,5,6,8]. These hormonal groups regulate distinct but interconnected metabolic pathways that ultimately determine plant performance under saline stress. ABA play a central role in osmotic adjustment and ion homeostasis through the regulation of ion transporters and osmoprotectant-related genes [9], while JA and ethylene contribute to the activation of antioxidant defense mechanisms and stress-signaling pathways [10,11]. In contrast, growth-promoting hormones sustain cell division, expansion and support carbon and nitrogen metabolism, thereby preserving photosynthesis, protein synthesis, and root system plasticity [12,13,14,15]. The coordinated hormonal control of these processes enables plants, including cereals, to reprogram their metabolism and maintain growth and productivity under saline conditions [16,17,18,19,20].

Barley (*Hordeum vulgare* L.) is a major cereal crop that ranks fifth worldwide in dry matter production. It serves as an important source of food, feed, and raw material for the malting industry and plays a key role in the agricultural systems of arid and semi-arid regions, including Tunisia [21]. Barley tolerance to salinity relies on several key mechanisms, including the maintenance of ion homeostasis through the regulation of sodium (Na^+^) and potassium (K^+^) levels, often through transporters such as HKT (high-affinity potassium transporters) and NHX (Na^+^/H^+^ antiporters) [22]. In addition, plant accumulates osmoprotectants such as proline and glycine betaine to protect cellular structures and enhance antioxidant defenses to mitigate oxidative stress. These responses are further regulated by transcription factors, making them potential targets for improving salt tolerance [23]. In this context, *H. maritimum*, a wild facultative halophyte native to coastal saline habitats but capable of growing under both non-saline and saline conditions, exhibits enhanced Na^+^ exclusion from leaves, osmotic adjustment through compatible solutes, and a greater antioxidant capacity compared to *H. vulgare*. In contrast, as a glycophyte, *H. vulgare* relies mainly on short-term osmotic adjustment and has a limited ability to control ion accumulation [3,21].

Beyond conventional of biotechnologically assisted plant breeding approaches, alternative strategies have been developed to mitigate the effects of adverse environmental conditions, including seed pretreatments with exogenous bioactive molecules involved in the regulation of plant responses to abiotic stress [20,24]. Seed priming is simple, low-cost and low-risk technique widely used to improve plant performance under stress conditions. It involves controlled seed hydration with chemical or natural compounds, followed by drying prior to germination, allowing metabolic activation without radicle emergence. This process enhances early vigor and stress preparedness through the activation of pre-germinative metabolic processes, including enzyme activation, antioxidant defense, osmotic adjustment, improved nutrient uptake and hormonal regulation mechanisms [25,26]. In barley, seed priming has been shown to enhance germination, early seedling vigor, and tolerance to abiotic stresses such as salinity and drought. Recent studies report that hydro-priming and chemical priming significantly improve emergence rate, photosynthetic performance, and grain yield under both optimal and stress conditions [19]. Therefore, seed priming represents a practical and sustainable agronomic strategy for barley cultivation, as it can be easily integrated into existing agricultural systems to enhance seedling establishment, field emergence, and yield stability, particularly in saline and marginal soils typical of arid and semi-arid regions [21].

Salicylic acid (SA) and calcium chloride (CaCl_2_) are increasingly recognized as effective signaling molecules involved in seed germination, early plant development, and stress tolerance mechanisms [27,28]. Both compounds act as priming agents that enhance plant resilience to salinity stress through physiological and biochemical modifications. SA is known to activate antioxidant defense systems and modulate hormonal pathways, while CaCl_2_, mainly through its calcium component, contributes to membrane integrity, ion balance, and intracellular signaling. Previous studies have shown that seed priming with SA or CaCl_2_ leads to improved germination, enhanced seedling growth, and reduced oxidative stress under saline conditions. For instance, priming barley seeds with SA and CaCl_2_ significantly improved growth parameters, decreased lipid peroxidation, and boosted antioxidant enzyme activity under salt stress [3]. Similarly, SA and CaCl_2_ were reported to mitigate sodium toxicity by enhancing potassium uptake and maintaining ionic balance in stressed barley seedlings [29]. In addition, seed priming with SA or CaCl_2_ has been shown to influence endogenous hormone levels such as ABA, IAA, and GAs [30,31], highlighting their potential role in hormonal regulation under salinity stress.

Monitoring crosstalk among hormonal pathways in plants is essential to clarify how seed priming influences stress-induced metabolic reprogramming and contributes to plant tolerance [32]. Although, great and considerable attention was paid to remediate hazardous impacts of salt stress on plants by applying diverse strategies, especially, among the cultivated species [33], information regarding the responses of spontaneous halophyte cultivated under adverse conditions remains limited. *H. maritimum* is a wild cereal naturally growing in saline environments, which excludes sodium from the leaves while accumulating proline and glycine betaine for osmotic adjustment, conferring a higher adaptive capacity to salinity than the domesticated *H. vulgare* [34,35].

Therefore, this study aimed to evaluate whether seed priming with salicylic acid (SA) or calcium chloride (CaCl_2_) can enhance salt tolerance in two barley species with contrasting adaptive capacities. We hypothesized that priming would (i) promote stress resilience by modulating specific hormone-related adaptive pathways; (ii) differentially affect the regulatory networks of the two species according to their inherent salt tolerance; and (iii) improve growth and physiological performance under saline conditions.

## 2. Results

### 2.1. Plant Biomass

**Figure 1:**
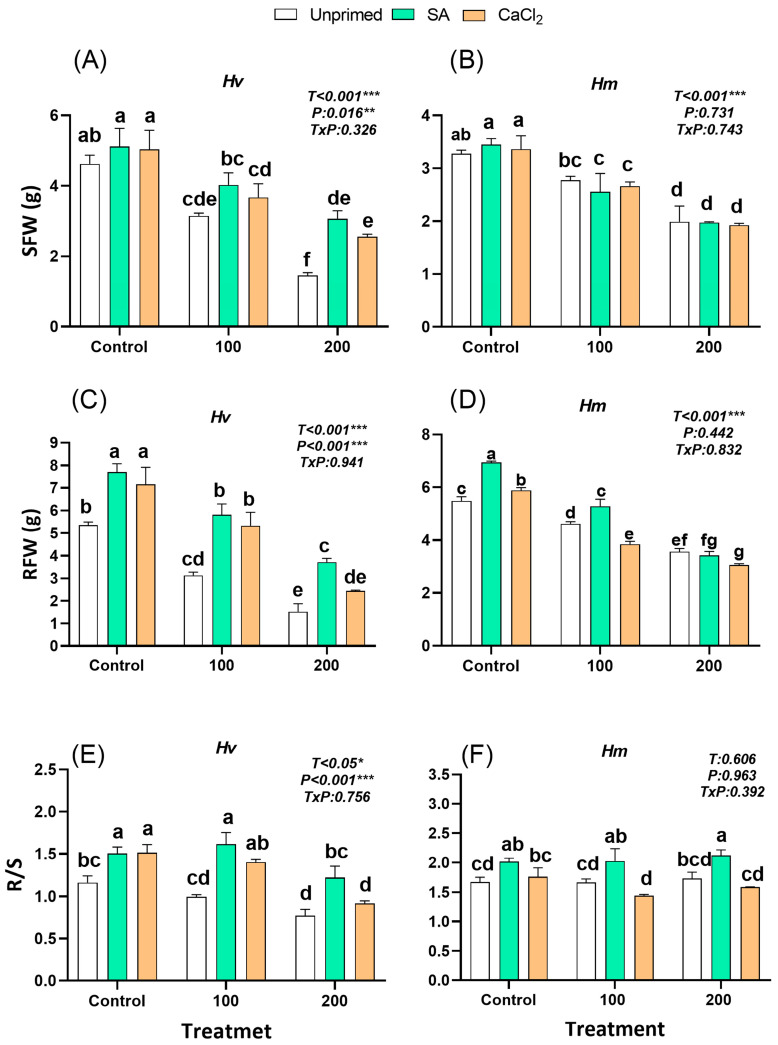
Effect of SA and CaCl_2_ seed priming on shoot fresh weight (SFW) in *Hv* (**A**) and *Hm* (**B**), root fresh weight (RFW) in *Hv* (**C**) and *Hm* (**D**) and root/shoot ratio (R/S) in *Hv* (**E**) and *Hm* (**F**) exposed to control conditions and two salinity levels (100 and 200 mM NaCl) for 15 days. Values are means of three replicates ± standard error. Different letters indicate significant differences among genotypes and treatments according to the Tukey test (*p* ≤ 0.05). Results of two-way ANOVA (*p* values reported) for salinity treatment (T), priming treatment (P), and their interaction (T × P) are indicated in the top right of each panel. *, **, and *** indicate statistically significant differences at *p* ≤ 0.05, *p* ≤ 0.01, and *p* ≤ 0.001, respectively.

Salinity reduced both shoot and root biomass in a concentration-dependent manner, but to a greater extent in the domesticated *Hv* (Figure 1A–D). Indeed, 200 mM NaCl reduced plant biomass by 69% in *Hv* and only 40.5% in *Hm*, compared to the control conditions. However, seed priming alleviated the negative impact of salinity on plant growth, especially in the less tolerant *Hv*, with SA showing a greater effect than CaCl_2_ (Figure 1A,C,E and Figure S1). The priming-mediated growth-promoting effect was higher in the root than in the shoot in both species, and was observed even in the absence of salinity. Indeed, the root/shoot ratio (R/S) increased in all the *Hv* primed plants, except those treated with CaCl_2_ under high salinity, while SA-pre-treatment increased this ratio in *Hm* plants regardless of the salt treatment (Figure 1E,F). The most significant priming effect was found in *Hv* plants derived from seeds pre-treated with SA and grown on a medium supplemented with 200 mM NaCl, resulting in a 2-fold increase in biomass compared to unprimed plants (Figure 1A–D). However, the positive effect of both seed priming agents in the wild *Hm* was observed only on roots of plants grown under control conditions, increasing biomasses by 41.5% and 32% of plants pretreated with SA and CaCl_2_, respectively, compared to unprimed ones. Under salinity, only SA increased root biomass by 27% in plants grown under 100 mM NaCl compared to their stressed unprimed equivalents, while CaCl_2_ had a negative effect in this species.

Overall, salinity reduces biomass, especially in the *Hv* species and SA and CaCl_2_ seed priming seems to promote growth in a stress level, organ, and species-dependent manner, but with a more significant positive effect under salinity in the less salt-adapted domestic barley. A principal component analysis (PCA) based on total fresh weight (TFW) further supported these findings, revealing distinct species-specific patterns. In *Hv*, priming under salinity (TFWsp) resulted in a clear shift from the unprimed salinity profile (TFWsu), suggesting a substantial recovery of growth. Interestingly, priming under control conditions (TFWp) led to a divergent response, indicating that its effect is context-dependent (Supplementary Figure S2A). Conversely, in *Hm*, all treatments clustered tightly, with minimal separation between control, salinity, and priming conditions. This further supports the idea that *Hm* exhibits a more stable growth phenotype, less dependent on external modulation and relying more on constitutive tolerance mechanisms (Supplementary Figure S2B).

### 2.2. Hormonal Profiling

#### 2.2.1. Salicylic Acid

SA, synthesized primarily in leaves and to a lesser extent in roots, and functions as a signaling molecule involved in the regulation of antioxidant defense, redox homeostasis, and stress-induced gene expression. In our study, endogenous SA levels increased under salt stress in both unprimed barley species, depending on the organ and salt concentration (Supplementary Figure S3A–E). In the domestic *Hv*, a similar 2 to 3-fold increase was found in the leaves under 100 and 200 mM NaCl, while no effect or a decrease was found in the roots (Supplementary Figure S3A,C,E). In the wild *Hm*, salinity progressively increases SA in both leaves and roots, reaching higher concentrations than in the domestic relative (Supplementary Figure S3B,D,E). SA priming increased SA concentration under control and, especially under the effect of 100 mM NaCl in leaves and roots of both species, compared to unprimed plants, with the highest differences found in the roots (Supplementary Figure S3A–E). However, under 200 mM NaCl, only a significant increase was found in the roots of *Hv*, while a decrease was observed in the leaves of *Hm*. CaCl_2_ priming increased leaf SA in both species under 100 mM NaCl to a similar extent as SA priming, while it inhibited the salt-induced SA accumulation in the leaves (Supplementary Figure S3). Overall, SA is differentially accumulated in both species in response to salinity, although a similar response was found to both priming agents. CaCl_2_ seems to interact with SA metabolism depending on the intensity of the stress.

#### 2.2.2. Abscisic Acid

**Figure 2:**
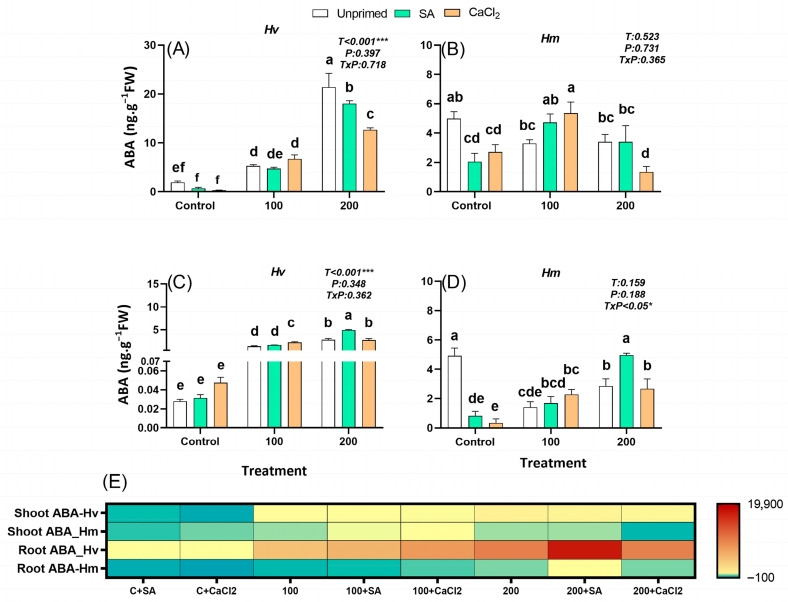
Effect of SA and CaCl_2_ seed priming on abscisic acid (ABA) endogenous content in leaves in *Hv* (**A**) and in *Hm* (**B**), and in roots in *Hv* (**C**) and in *Hm* (**D**) exposed to control conditions and two salinity levels (100 and 200 mM NaCl) during 15 days. Values are means of three replicates ± standard error. Different letters indicate significant differences among genotypes and treatments according to the Tukey test (*p* ≤ 0.05). Results of two-way ANOVA (*p* values reported) for salinity treatment (T), priming treatment (P), and their interaction (T × P) are indicated in the top right of each panel. * and *** indicate statistically significant differences at *p* ≤ 0.05 and *p* ≤ 0.001, respectively. The heatmap provides a complementary visualization of the data, representing the relative change (%) in hormone levels with respect to the unprimed, non-saline control for each species. This allows a clearer comparison of how salinity and priming treatments modify hormonal profiles in both species (**E**).

**Figure 3:**
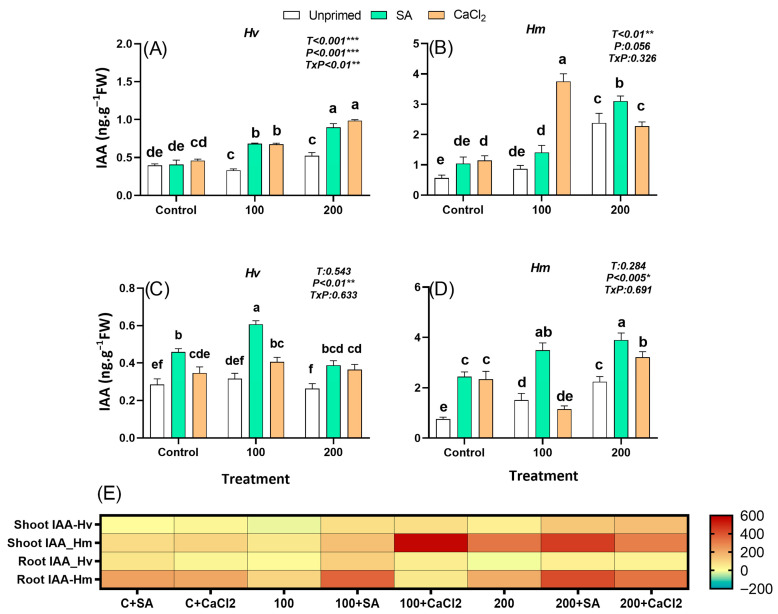
Effect of SA and CaCl_2_ seed priming on Indole-3-acetic acid (IAA) endogenous content in leaves in *Hv* (**A**) and in *Hm* (**B**), and in roots in *Hv* (**C**) and in *Hm* (**D**) exposed to control conditions and two salinity levels (100 and 200 mM NaCl) during 15 days. Values are means of three replicates ± standard error. Different letters indicate significant differences among genotypes and treatments according to the Tukey test (*p* ≤ 0.05). Results of two-way ANOVA (*p* values reported) for salinity treatment (T), priming treatment (P), and their interaction (T × P) are indicated in the top right of each panel. *, **, and *** indicate statistically significant differences at *p* ≤ 0.05, *p* ≤ 0.01, and *p* ≤ 0.001, respectively. The heatmap provides a complementary visualization of the data, representing the relative change (%) in hormone levels with respect to the unprimed, non-saline control for each species. This allows a clearer comparison of how salinity and priming treatments modify hormonal profiles in both species (**E**).

Abscisic acid (ABA), synthesized mainly in roots and leaves in response to osmotic stress, acts as a systemic signal that regulates stomatal closure, osmotic adjustment, and ion homeostasis. In our study, constitutive ABA levels were 2.6 (leaves) and 175.5 (roots) fold higher in the salt-adapted species *Hm* than in the domestic *Hv* in the absence of salt stress (Figure 2). Indeed, only *Hv* responded by accumulating ABA, particularly under high salinity, while *Hm* exhibited a decrease in ABA concentrations in both organs under both salinity levels, indicating a species-specific response (Figure 2A–E). Interestingly, SA priming reduced the constitutive ABA levels in both species, except in roots of *Hv* plants, where ABA levels remained unaffected (Figure 2A–E). At 100 mM NaCl, CaCl_2_ priming significantly increased ABA levels in the roots of *Hv* (Figure 2C) and the leaves of *Hm* (Figure 3B), while at 200 mM NaCl, SA priming led to a substantial increase in ABA content in the roots of both species (Figure 2C,D). In contrast, CaCl_2_ priming at this higher salinity regime reduced ABA levels in both roots and leaves across both species (Figure 2A–E). Overall, only the domestic *Hv* accumulated ABA in response to salinity, which was alleviated by priming, especially in leaves of plants grown from seeds pretreated with SA, while the salt-adapted species was insensitive to the stress in terms of the typical ABA accumulation. Both species exhibit similar responses to priming agents, which interact with ABA metabolism depending on salinity levels.

#### 2.2.3. Indole-3-Acetic Acid

**Figure 4:**
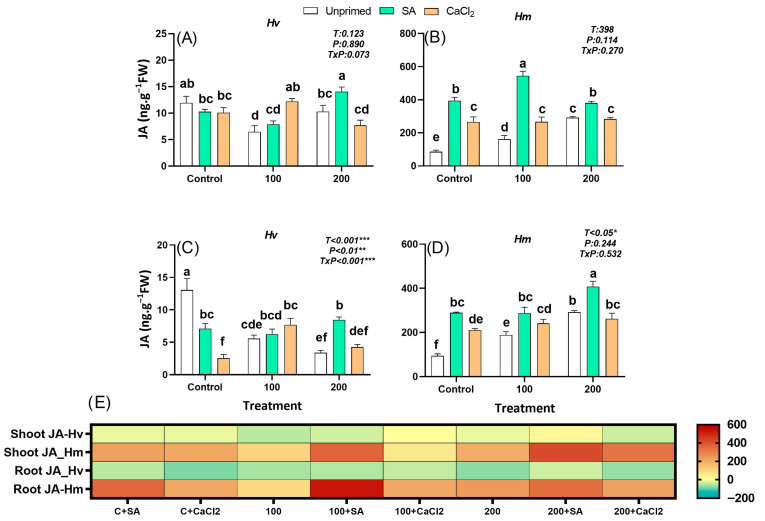
Effect of SA and CaCl_2_ seed priming on jasmonic acid (JA) endogenous content in leaves in *Hv* (**A**) and in *Hm* (**B**), and in roots in *Hv* (**C**) and in *Hm* (**D**) exposed to control conditions and two salinity levels (100 and 200 mM NaCl) during 15 days. Values are means of three replicates ± standard error. Different letters indicate significant differences among genotypes and treatments according to the Tukey test (*p* ≤ 0.05). Results of two-way ANOVA (*p* values reported) for salinity treatment (T), priming treatment (P), and their interaction (T × P) are indicated in the top right of each panel. *, **, and *** indicate statistically significant differences at *p* ≤ 0.05, *p* ≤ 0.01, and *p* ≤ 0.001, respectively. The heatmap provides a complementary visualization of the data, representing the relative change (%) in hormone levels with respect to the unprimed, non-saline control for each species. This allows a clearer comparison of how salinity and priming treatments modify hormonal profiles in both species (**E**).

IAA, primarily produced in young leaves and shoot apices and transported basipetally to roots, regulates root development and elongation. In this study, in the absence of salt treatments, constitutive IAA levels were 3-fold higher in roots of the salt-adapted species *Hm* than in the domesticated *Hv* (Figure 3C–E); however, in leaves, both barley species had similar levels (Figure 3A,B). In the wild *Hm*, salt stress progressively increases IAA concentrations in both organs, reaching higher concentration under 200 mM NaCl, where the content of this hormone was 3–4-fold higher compared to control conditions (Figure 3B,D,E). However, for the domestic *Hv*, constitutive IAA levels increased slightly only in leaves of plants subjected to 200 mM NaCl (37% compared to the control) (Figure 4A,C,E). Despite the treatment, priming generally tended to enhance ABA levels, with a more pronounced effect observed in *Hm* species (Figure 4A–E). Both priming agents exhibited a consistent pattern of ABA accumulation in domestic *Hv* species, significantly increasing ABA levels under salinity conditions compared to unprimed plants (Figure 3A,E). In the salt-adapted species *Hm*, priming with SA promoted the IAA accumulation in the leaves at 200 mM and in the roots at both 100 and 200 mM NaCl, while CaCl_2_ priming elevated IAA concentrations in the leaves at the low salinity regime and in the roots at the high salinity regime, relative to non-pre-treated counterparts (Figure 3B,D). Overall, an interaction seems to occur between IAA and priming that depends on both the priming agent and the species. Among the species studied, only the salt-adapted species *Hm* accumulated IAA in the roots in response to salinity, which was stimulated by priming in both species, but more intensively in SA-primed and particularly pronounced in the wild *Hm*.

#### 2.2.4. 1-Aminocylopropane-1-Carboxylic Acid

**Figure 5:**
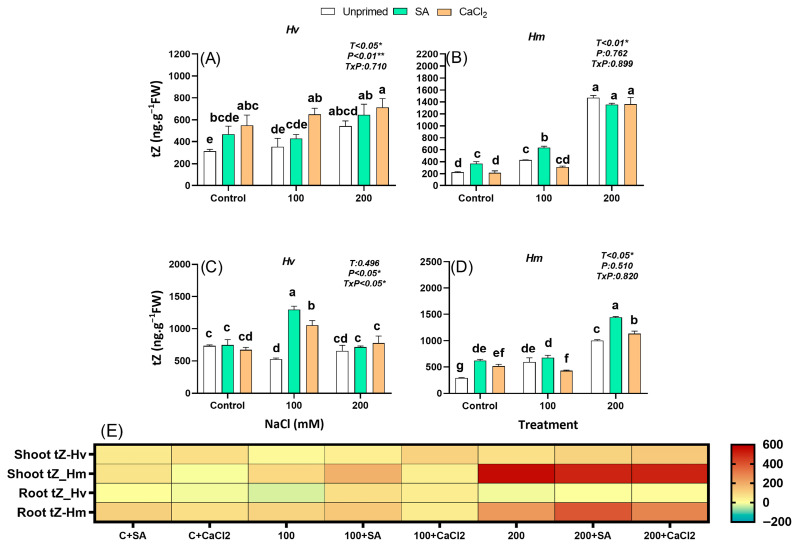
Effect of SA and CaCl_2_seed priming on trans-zeatin (tZ) endogenous content in leaves in *Hv* (**A**) and in *Hm* (**B**), and in roots in *Hv* (**C**) and in *Hm* (**D**) exposed to control conditions and two salinity levels (100 and 200 mM NaCl) during 15 days. Values are means of three replicates ± standard error. Different letters indicate significant differences among genotypes and treatments according to the Tukey test (*p* ≤ 0.05). Results of two-way ANOVA (*p* values reported) for salinity treatment (T), priming treatment (P), and their interaction (T × P) are indicated in the top right of each panel. * and ** indicate statistically significant differences at *p* ≤ 0.05 and *p* ≤ 0.01, respectively. The heatmap provides a complementary visualization of the data, representing the relative change (%) in hormone levels with respect to the unprimed, non-saline control for each species. This allows a clearer comparison of how salinity and priming treatments modify hormonal profiles in both species (**E**).

ACC is synthesized mainly in actively growing tissues, such as root tips, young leaves, and developing shoots, and serves as the immediate precursor of ethylene, a key gaseous hormone involved in stress signaling, growth regulation, and senescence. In this study, in the absence of both priming and saline stress, the concentration of ACC was significantly higher in the *Hm* species compared to the *Hv* one (Figure 5A–D). The presence of saline stress decreased the endogenous content of ACC in *Hm* species, particularly at 100 mM of NaCl (Supplementary Figure S4B,D,E). However, in the *Hv* species, ACC content remained stable under saline conditions, except in the roots at 100 mM NaCl, where it increased (Supplementary Figure S4A,C,E). In *Hv* species, SA pre-treatment significantly increased ACC levels in the leaves while maintaining stable concentrations in the roots across all salinity treatments (Supplementary Figure S4A,C,E). In contrast, CaCl_2_ pre-treatment preserved stable ACC levels in the leaves regardless of salinity regime, but induced an increase in roots under saline stress compared to non-pretreated plants (Supplementary Figure S4A,C,E). In *Hm* species, SA pre-treatment reduced ACC levels under control conditions but increased them under salinity in both roots and leaves compared to unprimed plants. CaCl_2_ pre-treatment inhibited ACC levels under non-stress conditions in both organs, with no effect observed under saline stress (Supplementary Figure S4B,D,E). Generally, ACC levels showed species-specific responses to salinity and priming: while *Hv* maintained relatively stable ACC levels under stress, *Hm* exhibited a decrease unless pre-treated, particularly with SA. Priming treatments modulated ACC content differently across species, organs, and salinity conditions.

#### 2.2.5. Jasmonic Acid

JA is mainly synthesized in leaves and acts as a key signaling molecule in the regulation of stress tolerance, antioxidant defense, and secondary metabolism. In our study, JA levels were 7 (leaves) and 6 (roots) fold higher in the salt-adapted species *Hm* than in the domestic *Hv*, under control conditions (Figure 4A–D). In the wild *Hm*, salinity gradually increased endogenous JA levels in both organs from unprimed barley plants, reaching higher concentrations than in the domestic relative. However, in the domestic *Hv*, a decrease was found in the leaves and the roots of plants grown under both salt concentrations (Figure 4A–E). Interestingly, SA priming increased JA concentration in the salt-adapted species *Hm* under both control and saline conditions, especially under the effect of 100 mM NaCl in leaves and the effect of 200 mM NaCl in roots compared to unprimed plants, with the highest differences found in the leaves (Figure 4B,D,E). Moreover, a significant increase in JA content was found in both leaves and roots of primed *Hv* plants subjected to 200 mM NaCl compared to unprimed ones (Figure 4A,C,E). CaCl_2_ priming promoted leaf and root JA in the wild *Hm* species under both control and 100 mM NaCl, while no effect was found in plants subjected to 200 mM NaCl, compared to unprimed plants and it was less effective than SA in increasing the accumulation of JA under 100 mM NaCl (Figure 4B,D). Moreover, CaCl_2_ improved constitutive JA levels in leaves and roots of *Hv* grown only under 100 mM NaCl compared to unprimed plants (Figure 4A,C). Overall, JA is differentially affected by salt in both species. Only the salt-adapted species accumulated JA in response to salinity, which was increased by priming. In contrast, domesticated *Hv* showed reduced JA levels under stress, but priming, particularly with SA, enhanced JA accumulation.

#### 2.2.6. Trans-Zeatin

tZ, one of the most active cytokinins, is synthesized mainly in root tips and transported to the shoot via the xylem, where it promotes cell division and chloroplast development. Under control conditions, tZ levels were 40% (leaves) and 151% (roots) times higher in the domestic *Hv* species compared to the salt-adapted *Hm*. Salinity gradually increased endogenous tZ concentration in the leaves of both unprimed barley species and in the roots of adapted *Hm* (Figure 5A–E). Both priming agents enhanced tZ levels in the roots in *Hv* at 100 mM NaCl and in *Hm* under control and high salinity (200 mM NaCl) conditions. SA priming significantly increased tZ levels in the leaves of *Hv* under all conditions, while CaCl_2_ pre-treatment enhanced tZ concentrations in the leaves of *Hm* under control and 100 mM NaCl compared to unprimed plants (Figure 5A–E). Overall, tZ is differentially accumulated in both species in response to salinity but to a greater extent in the wild *Hm* than in the domestic relative. Priming treatments showed species-specific effects, with CaCl_2_ being more effective in *Hv* leaves, while *Hm* responded more strongly under specific conditions.

#### 2.2.7. Zeatin Riboside

**Figure 6:**
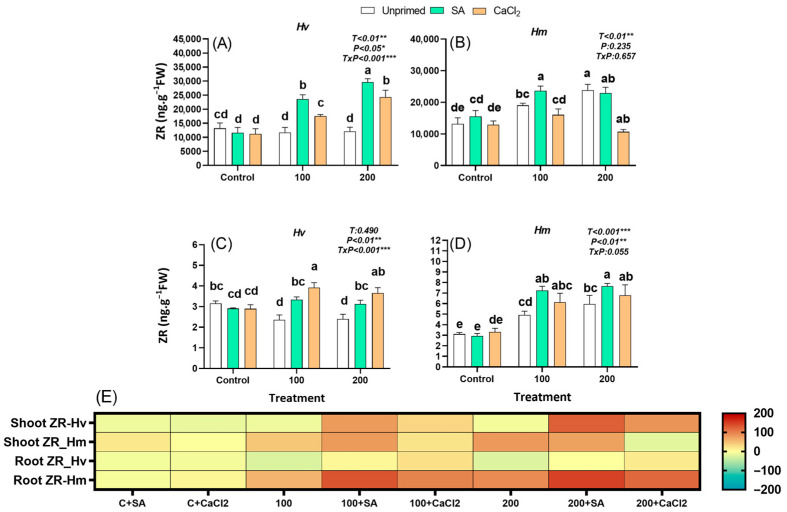
Effect of SA and CaCl_2_ seed priming on zeatin riboside (ZR) endogenous content in leaves in *Hv* (**A**) and in *Hm* (**B**), and in roots in *Hv* (**C**) and in *Hm* (**D**) exposed to control conditions and two salinity levels (100 and 200 mM NaCl) during 15 days. Values are means of three replicates ± standard error. Different letters indicate significant differences among genotypes and treatments according to the Tukey test (*p* ≤ 0.05). Results of two-way ANOVA (*p* values reported) for salinity treatment (T), priming treatment (P), and their interaction (T × P) are indicated in the top right of each panel. *, **, and *** indicate statistically significant differences at *p* ≤ 0.05, *p* ≤ 0.01, and *p* ≤ 0.001, respectively. The heatmap provides a complementary visualization of the data, representing the relative change (%) in hormone levels with respect to the unprimed, non-saline control for each species. This allows a clearer comparison of how salinity and priming treatments modify hormonal profiles in both species (**E**).

ZR is a mobile cytokinin form synthesized mainly in the roots as a conjugated derivative of trans-zeatin. It serves as a transport and temporary storage form that moves through the xylem to the shoots, where it can be reconverted into the biologically active tZ. In our study, even though both species registered similar constitutive ZR levels in the absence of salt stress, only unprimed *Hm* species responded by accumulating ZR in both organs under salinity, while this hormone was not affected in leaves and decreased in roots of *Hv*, under both salt concentrations (Figure 6A–E). Priming with both agents did not affect ZR under optimal conditions in both barley species. In the domestic *Hv* plants subjected to salinity stress, both SA and CaCl_2_ priming significantly increased ZR levels in both leaves and roots (Figure 6A–C). In the salt-adapted *Hm*, SA stimulated ZR concentration in the leaves only under 100 mM NaCl and in roots under both salinity regimes. However, CaCl_2_ priming led to a significant decrease in ZR in leaves at 200 mM NaCl and had no effect in the other conditions/organs (Figure 6B,D). Overall, only salt-adapted *Hm* accumulated ZR in response to salinity, which was promoted by SA priming, while the domestic species was insensitive to the stress in terms of the typical ZR accumulation unless primed. An interaction seems to occur between ZR and priming, particularly in *Hm* species.

#### 2.2.8. Isopentenyl Adenine

**Figure 7:**
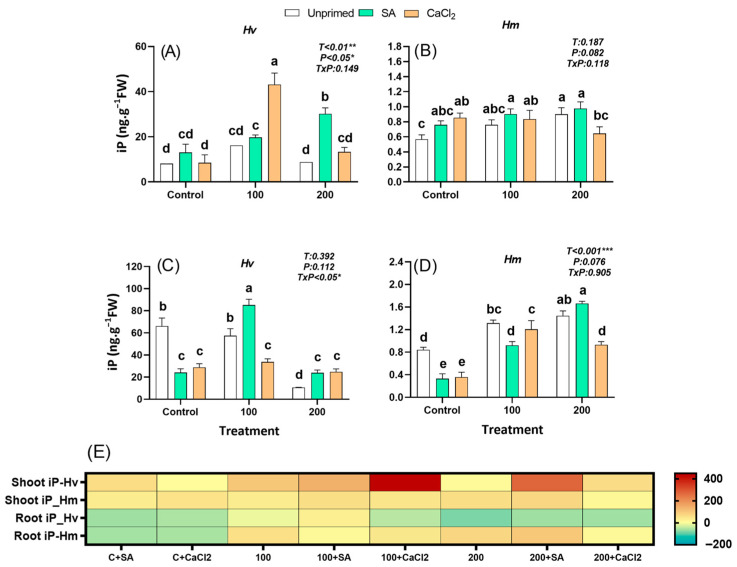
Effect of SA and CaCl_2_ seed priming on isopentenyl adenine (iP) endogenous content in leaves in *Hv* (**A**) and in *Hm* (**B**), and in roots in *Hv* (**C**) and in *Hm* (**D**) exposed to control conditions and two salinity levels (100 and 200 mM NaCl) during 15 days. Values are means of three replicates ± standard error. Different letters indicate significant differences among genotypes and treatments according to the Tukey test (*p* < 0.05). Results of two-way ANOVA (*p* values reported) for salinity treatment (T), priming treatment (P), and their interaction (T × P) are indicated in the top right of each panel. *, **, and *** indicate statistically significant differences at *p* ≤ 0.05, *p* ≤ 0.01, and *p* ≤ 0.001, respectively. The heatmap provides a complementary visualization of the data, representing the relative change (%) in hormone levels with respect to the unprimed, non-saline control for each species. This allows a clearer comparison of how salinity and priming treatments modify hormonal profiles in both species (**E**).

IP, a cytokinin synthesized both in roots and developing shoots, regulates early cell division and shoot initiation and their levels were less abundant than ZR and tZ in the different organs of plants of both species, regardless of salt treatment. Indeed, constitutive iP levels were 1332% (leaves) and 7769% (roots) fold higher in the domestic *Hv* than in the salt-adapted species *Hm*, under control conditions (Figure 7A–E). Under salinity, only unprimed *Hm* responded by accumulating iP in both organs, while this hormone remained stable (leaves) or decreased (roots) in unprimed *Hv* species (Figure 7A–E). The iP levels increased gradually in the wild species to reach 50% in leaves and 75% in roots under 200 mM NaCl compared to the control (Figure 7B,D,E). Under favourable conditions, seed priming had an advantageous effect only in leaves of the salt-adapted *Hm* species (Figure 7B). Under a restrictive environment, SA stimulated Ip levels in roots of *Hm* plants under 200 mM NaCl compared to unprimed plants (Figure 7D). In the domestic *Hv*, SA priming significantly increased iP concentration in leaves of plants grown under 200 mM NaCl by 130% and in roots of plants grown under 100 mM NaCl by 50% compared to unprimed plants (Figure 7A,B). However, CaCl_2_ priming improved constitutive iP levels only in leaves of *Hv* plants grown under 100 mM NaCl, showing an increase of 166% compared to unprimed plants, whereas it decreased or had no effect on iP concentration in *Hm* plants regardless of salt levels (Figure 7A–D). Overall, only the salt adapted species accumulated iP in response to salinity, with priming having no effect in leaves and an inhibitory effect in the roots. In contrast, the domestic species was insensitive to the stress in terms of the typical iP accumulation and required priming to enhance iP levels under stress.

#### 2.2.9. Gibberellin A4

**Figure 8:**
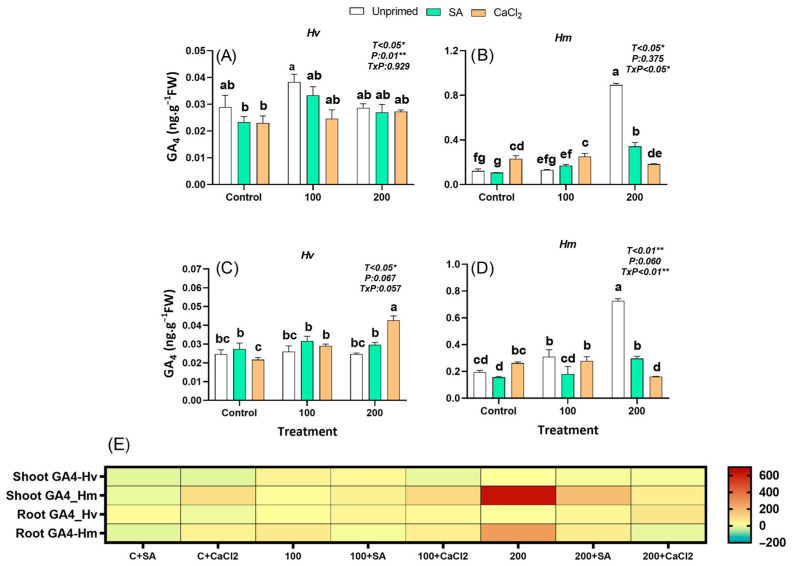
Effect of SA and CaCl_2_ seed priming on Gibberellin A_4_ (GA_4_) endogenous content in leaves in *Hv* (**A**) and in *Hm* (**B**), and in roots in *Hv* (**C**) and in *Hm* (**D**) exposed to control conditions and two salinity levels (100 and 200 mM NaCl) during 15 days. Values are means of three replicates ± standard error. Different letters indicate significant differences among genotypes and treatments according to the Tukey test (*p* < 0.05). Results of two-way ANOVA (*p*-values reported) for salinity treatment (T), priming treatment (P), and their interaction (T × P) are indicated in the top right of the panel. * and ** indicate statistically significant differences at *p ≤* 0.05 and *p ≤* 0.01, respectively. The heatmap provides a complementary visualization of the data, representing the relative change (%) in hormone levels with respect to the unprimed, non-saline control for each species. This allows a clearer comparison of how salinity and priming treatments modify hormonal profiles in both species (**E**).

GA_4_ is synthesized mainly in young, actively growing tissues such as shoot apices, expanding leaves, and developing seeds and promote stem elongation, leaf expansion, and seed germination by stimulating cell elongation and division. Under salinity, GA biosynthesis is often inhibited, leading to reduced growth as part of the trade-off between stress defense and development. In the present study, under control conditions, GA4 levels were 322% (leaves) and 693% (roots) times lower in the domestic *Hv* species compared to the salt-adapted *Hm* (Figure 8). Only unprimed *Hm* species responded by accumulating gibberellin GA4 in leaves of plants grown under 200 mM NaCl and in roots of plants grown under both 100 mM and 200 mM NaCl, while this hormone was not affected or decreased in both organs of the domestic species, regardless of the salt concentrations (Figure 8A–E). SA priming reduced or had no effect on GA_4_ concentrations in both organs of stressed plants of both barley species (Figure 8A–D). However, CaCl_2_ significantly improved GA_4_ concentrations in leaves (control and 100 mM NaCl) of *Hm* plants and in roots of the domestic *Hv* grown under 200 mM NaCl compared to unprimed plants (Figure 8B,C,E). Overall, only the salt adapted species responded to salinity with increased GA_4_ accumulation, particularly in roots, unlike *Hv*, which showed no response. SA priming had minimal impact on GA_4_ levels, while CaCl_2_ selectively enhanced GA_4_ in specific organs and conditions, suggesting a species- and treatment-dependent regulation of gibberellin dynamics.

### 2.3. Correlation and Principal Component Analyses

#### 2.3.1. Pairwise Correlations Across Treatments and Species

Under control conditions, a significant negative correlation was found between root to shoot ratio (R/S) and shoot SA levels in *H. vulgare*, whereas in *H. maritimum*, this ratio negatively correlated with root SA (Supplementary Figure S5A). In primed plants, both shoot and root SA were positively associated with RFW and R/S exclusively in *Hm* (Supplementary Figure S5B). When salinity was applied (without priming), shoot and root SA were negatively correlated with both shoot (root SA) and Root FW (root and shoot SA) only in *Hm* (Supplementary Figure S5C). However, under salinity with priming, SA accumulation, particularly in shoots, was positively correlated with SFW, RFW, and R/S in both species (Supplementary Figure S5D).

Neither non-stressed *Hv* nor *Hm* exhibited significant correlations between ABA levels and biomass parameters, irrespective of priming pre-treatment (Supplementary Figure S5A,B). However, salinity triggered strong negative correlations in *Hv* between ABA concentrations (in both tissues) and all growth parameters. In contrast, *Hm* displayed a more limited response with only root ABA levels negatively correlated with SFW and RFW, while both shoot and root ABA showed weak, non-significant positive associations with R/S (Supplementary Figure S5C). Notably, priming preserved these negative correlations in *Hv* shoots, attenuated them in *Hm* roots, and shifted shoot ABA in *Hm* toward a positive correlation with shoot and root fresh weight under salinity (Supplementary Figure S5D).

Correlational analyses of IAA with growth parameters revealed tissue- and condition-specific roles in both *Hv* and *Hm*. In non-stressed *Hv*, root IAA levels negatively correlated with SFW (Supplementary Figure S5A). Under salinity, shoot IAA in *Hv* consistently exhibited negative correlations with SFW, RFW, and R/S, regardless of priming. Conversely, root IAA in *Hv* displayed significant positive correlations with all growth parameters, particularly significant when priming was applied (Supplementary Figure S5C,D). In salinized *Hm*, both shoot and root IAA maintained negative associations with RFW and SFW, but in root, IAA were more nuanced. However, upon priming, root IAA in this species was negatively correlated with SFW but positively associated with R/S (Supplementary Figure S5C,D).

The ethylene precursor, ACC, also showed species-specific trends. In unprimed *Hm* grown under control conditions, root ACC negatively correlated with SFW (Supplementary Figure S5A). Under salinity, shoot ACC in both species exhibited strong negative correlations with SFW and RFW, while root ACC was positively associated with biomass in *Hv* and negatively in *Hm* (Supplementary Figure S5C). Under saline conditions with priming, shoot and root ACC in *Hm* displayed positive correlations with RFW and R/S, while in *Hv*, only shoot ACC correlated positively with R/S (Supplementary Figure S5D).

JA levels were positively associated with RFW in the root of *Hm* under control conditions, and this effect was intensified with priming, extending to both shoot and root JA in *Hm* and to shoot JA in *Hv* (Supplementary Figure S5A,B). Under salinity without priming, both species exhibited patterns similar to those observed with ACC and IAA: shoot JA was negatively correlated with biomass in both species, while root JA correlated positively and negatively with growth in *Hv* and *Hm*, respectively (Supplementary Figure S5C). Upon priming, only *Hm* displayed a positive association between JA concentrations in both organs and RFW (shoot JA) and R/S (shoot and root JA) (Supplementary Figure S5D).

tZ dynamics varied also between species in terms of correlations with biomass parameters. Under non-stressed conditions, in unprimed *Hv* plants, shoot tZ correlated negatively with R/S, while in primed *Hm*, tZ (both shoot and root) was positively associated with RFW and R/S (Supplementary Figure S5A,B). Salinity led to negative correlations between tZ and growth parameters in both species Supplementary (Figure S5C). Following priming, these negative correlations persisted, except for root tZ in *Hv*, which shifted to a positive correlation with all biomass traits (Supplementary Figure S5C).

In *Hv* species, shoot and root ZR correlated positively and negatively with SFW and RFW, respectively, under control conditions (Supplementary Figure S5A). In *Hm*, salinity induced a significant negative correlation between shoot ZR and RFW (Supplementary Figure S5C); however, this relationship shifted to a positive correlation under priming (Supplementary Figure S5D). Moreover, under priming, both shoot and root ZR levels in *Hm* were positively associated with R/S, indicating a priming-enhanced role of ZR in promoting root allocation under stress (Supplementary Figure S5D).

Under control conditions with priming, iP concentrations measured in the shoot exhibited a negative correlation with RFW in *Hv*, while a positive correlation with R/S was observed in *Hm* (Supplementary Figure S5B). Under salinity, *Hv* exhibited strong positive correlations between both shoot and root iP levels and all growth parameters, a pattern not observed in *Hm* (Supplementary Figure S5C). When salinity was combined with priming, *Hv* maintained positive correlations only with root iP, while *Hm* remained unresponsive to the treatment in terms of correlations (Supplementary Figure S5D).

Gibberellin A_4_ exhibited a complex, species-specific correlation pattern. In primed *Hm* under control conditions, both shoot and root GA_4_ levels were negatively associated with RFW, while only shoot GA_4_ showed a negative correlation with R/S (Supplementary Figure S5B). Under salinity, *Hv* displayed consistent positive correlations between shoot GA_4_ and all growth parameters. In contrast, *Hm* showed negative correlations between GA_4_ levels in both organs and shoot and root biomass (Supplementary Figure S5C). Under salinity combined with priming, root GA_4_ in *Hv* shifted to a negative correlation with growth traits (Supplementary Figure S5D).

In summary, under salinity without priming, *Hv* showed negative correlations between biomass and several shoot hormones (ABA, IAA, ACC, JA, tZ), while root ACC, JA, iP, and shoot GA_4_ correlated positively with growth. In contrast, *Hm* exhibited predominantly negative associations between most hormones and biomass across both tissues. Upon priming, *Hv* maintained positive correlations particularly with root-derived signals (IAA, tZ, iP), while *Hm* exhibited a shift toward positive associations between several hormones—especially shoot-derived signals (SA, ACC, JA, ZR)—and growth parameters, particularly RFW and R/S.

#### 2.3.2. Principal Component Analysis

Principal component analysis was performed to further explore the multivariate relationships among hormonal and biomass variables across species and treatments, with a particular focus on the salinity conditions (Supplementary Figures S6 and S7). The results were consistent with the correlation analysis, confirming several treatment- and tissue-specific hormone-growth associations.

In *Hv* species, under salinity conditions, RFW, SFW, and R/S covaried along PC1, which accounted for 68.9% of the total variability. These growth-related traits clustered with several root hormones, including ACC, IAA, iP, and JA, as well as shoot-derived SA, iP, and GA_4_. In contrast, most shoot hormones (ABA, tZ, ACC, IAA, and JA), together with ABA and tZ in the root, formed a separate cluster in the opposite direction, suggesting a hormonal profile associated with reduced biomass accumulation (Supplementary Figure S6A). Upon priming, the PCA structure shifted markedly in *Hv* plants subjected to salinity, where PC1 and PC2 explained 49.6% and 22% of the variance, respectively. Growth-related variables (SFW, RFW, R/S) remained grouped with several root hormones, including, IAA, tZ, SA, and iP, clustered on the positive side of PC1, along with shoot SA, and to a lesser extent, shoot GA_4_ and ACC. In contrast, the negative side of PC1 included shoot tZ, IAA, and ABA, as well as root ABA and GA_4_, pointing to a contrasting hormonal signature more consistent with stress signaling than with growth promotion (Supplementary Figure S6B).

In *Hm* plants subject to salinity, PCA showed that PC1 accounted for 94.3% of the total variance, while PC2 explained only 5.6%. Most hormonal variables from both root and shoot covaried along the positive side of PC1, forming a tight cluster. The root-to-shoot ratio and shoot ABA also covaried positively with PC1, although they were located slightly apart from the core hormonal group. In contrast, RFW and SFW covaried negatively with PC1 and were positioned on the negative side of PC1, with an orthogonal distribution relative to the hormone-associated variables. This orthogonal distribution indicates a pronounced decoupling between growth performance and hormonal profiles under these conditions (Supplementary Figure S7A). Following seed priming, PC1 and PC2 explained 37.7% and 32.3% of the total variance, respectively, indicating a more distributed and complex response compared to the unprimed condition. RFW and SFW covaried in the lower left quadrant of the PCA plot, along with several shoot hormones (ABA, SA, JA, ACC, ZR, and iP), as well as root SA and ACC. These variables formed a coherent group along similar loadings on both principal components. The R/S ratio was closely grouped with root IAA, JA, and ZR, indicating a coordinated shoot–root hormonal adjustment associated with root investment. Most growth-promoting hormones from both shoots (GA_4_, IAA, tZ) and roots (ZR, iP, GA_4_, tZ) remained in separate clusters from biomass (RFW, SFW), suggesting their variation was not the primary driver of total biomass increase but may contribute to allocation patterns (Supplementary Figure S7B).

Overall, PCA revealed marked differences in hormone–growth coordination strategies between the less salt-tolerant *Hv* and the salt-adapted *Hm*. In *Hv*, salinity separated growth traits from a cluster of shoot-derived stress hormones, whereas priming realigned biomass with root-derived growth-promoting hormones (IAA, tZ, iP), consistent with recovery of growth potential. In *Hm*, salinity showed a clear decoupling between biomass and hormonal profiles, reflecting growth maintenance largely independent of dynamic hormone–growth coupling. Priming produced a more diversified variance structure and modestly increased alignment between certain hormones (SA, ACC, JA, ZR) and growth traits, but without the strong growth integration observed in *Hv*.

## 3. Discussion

Salinity significantly impairs biomass accumulation in barley, although the extent and underlying mechanisms differ between the domesticated *Hordeum vulgare* and the wild *Hordeum maritimum*. In our study, exposure to 200 mM NaCl reduced biomass by 69% in *Hv* compared with 40.5% in *Hm*, highlighting the intrinsic salt tolerance of the wild relative (Figure 1). This contrast reflects distinct strategies: *Hv* seems to require inducible adjustments, while *Hm* appears to rely on constitutive mechanisms such as high antioxidant capacity, stable osmolyte accumulation, and hormonal homeostasis [3,36]. In our study, under non-primed conditions, *H. vulgare* showed stress-induced ABA accumulation and reduced levels of growth-promoting hormones (IAA, CKs), while *H. maritimum* maintained a stable hormonal balance with high constitutive levels of these hormones, supporting its greater tolerance. Similar constitutive mechanisms have been widely described in halophytic barley and other wild relatives adapted to saline environments [37,38]. The observed decline in growth is consistent with the known effects of salt stress in cereals, where the accumulation of sodium in the cell walls and cytoplasm of leaves, particularly at high salinity levels, disrupts water balance, ion homeostasis, and hormonal signaling [19]. Seed priming with SA and CaCl_2_ significantly improved the growth of barley plants exposed to salinity compared with plants derived from unprimed seeds. This improvement depended on salt concentration and pretreatment agent, with responses differing between species and tissues. Among the two priming agents, SA consistently produced a stronger growth-promoting effect than CaCl_2_, likely because SA, as a phytohormone, directly regulates growth and activates complex defense and signaling pathways, whereas CaCl_2_ mainly acts as an essential nutrient and secondary messenger mitigating ionic and osmotic stress. Likewise, among the two barley species, the priming effect was particularly pronounced in *Hv*, where SA doubled biomass under high salinity, largely through stimulation of root growth and an increased root-to-shoot ratio (Figure 1C,E), highlighting its efficacy in enhancing stress resilience via improved growth, photosynthetic performance, ionic homeostasis, and hormonal regulation [3]. In contrast, *Hm* displayed only modest benefits from priming, and CaCl_2_ caused a reduction in root growth at higher salinity levels (Figure 1B,D,F), likely because its strong constitutive tolerance mechanisms made the combined ionic and metabolic stimulus of priming excessive for this halophytic species. These findings suggest that *Hv* is more responsive to priming-induced physiological adjustments, whereas *Hm*, with its constitutive tolerance, benefits less from external modulation. This differential response also supports the general ecological and physiological principle that organisms with greater environmental plasticity or sensitivity often benefit most from environmental enhancements or attenuators, whereas resilient, pre-adapted specialists derive less relative advantage from the same interventions [39].

Priming agents are generally known to stimulate plant physiological responses, particularly those related to primary metabolism [40]. In agreement with our results, exogenous SA application has been shown to improve plant biomass and seed yield in soybean plants by mitigating the adverse effects of salinity, likely through counteracting NaCl-induced nutrient imbalances [41]. Thus, the growth-promoting effect of SA observed especially in *Hv* may reflect improved nutrient homeostasis. Similarly, CaCl_2_ pretreatment in *Oryza sativa* has been reported to enhance starch hydrolysis and stimulate sugar availability for embryo feeding, leading to more vigorous seedlings and improved biomass, yield, and seed quality. Collectively, these comparisons support the interpretation that SA priming effectively restores growth in *Hv*, whereas the inherently tolerant *Hm* displays limited responsiveness. Phytohormones are recognized as the main endogenous regulators playing a key role in the ability of plants to adapt to abiotic stresses and to improve crop tolerance. In this context, ref. [42] emphasized that phytohormones modulate physiological, biochemical and molecular responses that are essential for plant survival. Consistent with this view, our results showed that salinity remarkably altered the hormonal status in leaves and roots of both barley species. Indeed, salinity-induced growth reduction has been widely associated with altered hormonal balance [6]. Hormonal profiling further revealed fundamental differences in the regulatory strategies of *Hv* and *Hm* under salinity and priming. In *Hv*, salinity triggered a marked accumulation of ABA in both roots and shoots, highlighting a reactive stress-signaling role. This was further supported by PCA, where shoot ABA clustered opposite to biomass-related traits in *Hv* under salinity, consistent with a stress-associated hormonal profile (Supplementary Figure S6A). However, priming, particularly SA, reduced ABA levels in leaves while maintaining them in roots (Figure 2A,C and Figure S8), consistent with reports showing that alleviating stress-induced ABA buildup is associated with improved salt tolerance in cereals [43,44]. In contrast, *Hm* registered high constitutive ABA content that decreased under stress (Figure 2A,C), suggesting a tolerance mechanism independent of stress-induced ABA accumulation. This is consistent with evidence indicating that modulation of ABA dynamics, rather than sustained accumulation, can contribute to improved salt tolerance [45].

IAA and JA were also differentially regulated, which further illustrates the contrasting strategies of both species. In *Hm*, IAA and JA were constitutively abundant in roots (IAA and JA) and leaves (JA) and further increased under salinity (Figure 4B,D and Figure 6B,D). This hormonal profile suggests a pre-adapted hormonal background that integrates growth and defense functions, thereby sustaining biomass under stress. In contrast, *Hv* showed only minor increases in IAA and a reduction in JA under salinity, patterns associated with impaired growth. Notably, priming, particularly with SA, reactivated both pathways in *Hv*, enhancing IAA levels and restoring JA (Figure 3A,C and Figure 4A,C). Importantly, in *Hv*, root-derived IAA correlated positively with SFW, RFW and R/S, underscoring its central role in priming-induced growth recovery (Supplementary Figure S5D). These patterns suggest that SA priming promotes a coordinated rebalancing of hormonal signaling, strengthening SA–IAA cross-talk that primarily drives root growth recovery, while SA–JA interactions play a role in activating complementary defense pathways and partially contributing to overall stress adaptation [46].

Regarding ACC, in *Hv*, salinity induced its accumulation in shoots (Supplementary Figures S4A,C and S8), which correlated negatively with biomass, and these associations disappeared under priming (Supplementary Figure S5). Although the role of ethylene in abiotic stress responses remains debated, our results are consistent with studies pointing to a negative role of its immediate precursor ACC in regulating plant growth under stress conditions, including salinity [4,5,47,48]. In contrast, *Hm* exhibited high constitutive ACC that declined under salinity (Supplementary Figure S4B,D), a trend that, similar to its ABA profile, suggests a tolerance mechanism independent of stress-induced hormonal accumulation, thereby avoiding growth inhibition typically associated with ethylene signaling.

Cytokinins provided further evidence of divergent regulatory strategies. While *Hv* exhibited higher basal levels of CKs such as tZ and iP, only *Hm* accumulated CKs (tZ, ZR, iP) in response to salinity (Figure 5, Figure 6, Figure 7 and Figure S8). By contrast, *Hv* required priming to stimulate CKs accumulation, mainly in roots, which was associated with improved root growth and increased R/S (Figure 5, Figure 6, Figure 7 and Figure S8). Under priming, root tZ and iP, similar to IAA, were positively correlated with SFW, RFW, and R/S (Supplementary Figure S5), highlighting these growth-promoting hormones as key positive signals driving biomass recovery and enhanced root allocation. This relationship was further supported by PCA, where growth traits clustered with these root-derived CKs and IAA, confirming their central role in biomass restoration (Supplementary Figure S6B). These findings suggest that *Hm* mobilizes Cks as part of its constitutive tolerance strategy, whereas *Hv* relies on priming-induced reprogramming of cytokinin metabolism to restore growth under stress [49,50]. Similar hormonal reprogramming has been reported in *Zea mays* and lentil. In maize, SA treatments at the seedling stages likewise ameliorate salt effects by modulating osmolytes, antioxidant enzymes and hormonal cross-talk (e.g., reducing excessive ABA signaling while supporting growth regulators), consistent with our finding that priming redirects hormonal networks toward root growth and ion/homeostasis benefits [51]. Recent studies have also reported that seed priming with SA enhances salt stress tolerance by boosting antioxidant defense in *Phaseolus vulgaris,* with larger proportional gains often observed in sensitive cultivated genotypes versus tolerant wild relatives. This pattern parallels our observation that *Hv* benefits more from priming than *Hm* [27]. Similarly, GA_4_ exhibited species-specific responses, with *Hm* increasing GA_4_ under salinity, whereas *Hv* required CaCl_2_ priming to enhance GA_4_ levels, suggesting that priming selectively modulates growth-related hormones to support stress adaptation. Taken together, these results indicate that the priming-mediated reduction of ABA, combined with increased root IAA and cytokinin levels, was the main hormonal adjustment supporting growth recovery and enhanced root allocation in *Hv* under salinity. These effects are consistent with observations in other crops, where SA priming limits reactive oxygen species, preserves photosynthetic capacity, restores nutrient balance, and rebalances stress versus growth signaling under salinity, including rice, maize, lentil, tomato, and quinoa [27,51,52,53]. Similar mechanistic patterns appear in rice, where exogenous SA enhances antioxidant defenses, reduces Na^+^ toxicity, and preserves photosynthetic capacity, often accompanied by shifts in ABA/auxin balance that favor growth under moderate salinity [54,55].

Collectively, all these findings underscore the complex interplay between species-specific hormonal regulation and growth responses under salinity, showing that *Hm* relies on constitutive tolerance mechanisms characterized by high basal hormone levels, whereas *Hv* benefits from priming-induced modulation of hormonal and physiological pathways (Supplementary Figure S8). Priming with SA, and to a lesser extent with CaCl_2_, contributes to re-establishing hormonal homeostasis under salinity by attenuating the excessive accumulation of ABA while simultaneously promoting growth-related hormones such as auxins, GAs, and CKs. SA may act as a signaling molecule that modulates stress responses and enhances antioxidant activity, thereby favoring growth and resilience, while Ca^2+^ supplied through CaCl_2_ may stabilize membranes and function as a secondary messenger in hormonal crosstalk, indirectly supporting ABA regulation and stomatal control. These complementary mechanisms were particularly evident in the less tolerant and plastic *H. vulgare*, in which priming led to the greatest growth improvement. This highlights the broad applicability of SA/Ca-based priming as a low-cost mitigation strategy and the potential of combining inducible priming with wild-relative traits to enhance crop resilience and productivity under environmental constraints.

## 4. Materials and Methods

### 4.1. Plant Material

Two barley species were used in this study: *Hordeum vulgare* cv. Manel provided by the National Institute of Agronomy of Tunis (INAT) and *Hordeum maritimum* were collected from Kalbia Sebkha (in Tunisia, which covers 8000 hectares in Sousse governorate at 35°’50′34″ N, 10°’16′18″ E South of Kondar).

### 4.2. Experimental Design and Growth Conditions

Before starting the experiment, seeds of each species were sterilized for 8 min with sodium hypochlorite (2%, *v*/*v*), thoroughly rinsed with distilled water and then soaked for 20 h either in distilled water (Unprimed), SA (Primed treatment with 1.25 mM of SA) or CaCl2 (Primed treatment with 5 mM of CaCl_2_) solution. The concentrations and times of the priming agents were chosen based on preliminary test, later confirmed by a comparison with previous investigations, such as in sorghum (*Sorghum bicolor* L.) and barley (*Hordeum maritimum* and *Hordeum vulgare*). For the germination assay, ten Petri dishes per species were prepared each containing 50 seeds placed on a double layer of moistened filter paper and incubated at 20 °C during 7 days in darkness. Homogenous seedlings were transferred to 5 L containers and grown for 7 days with half-strength aerated nutrient solution, followed by 15 days in full-strength solution. Plants were maintained in a growth chamber under controlled conditions (a day/night cycle of 16/8 h, 24 °C/18 °C regimes, PPFD of 200 mmol m^−2^ s^−1^ and 70% relative humidity). Plants were distributed into three groups according to the priming treatments (unprimed, SA and CaCl_2_). Each group was composed of nine containers, each containing eight plants. Each treatment included three biological replicates (containers), and each measurement represented the mean of three technical replicates. When plants reached the third leaf stage, two salinity levels (100 and 200 mM NaCl) were applied by adding NaCl to the nutrient solution. A control group without NaCl was maintained under the same conditions. These concentrations correspond to the salinity levels previously reported to induce measurable stress responses in barley without causing irreversible damage, allowing the assessment of both growth inhibition and adaptive physiological mechanisms [21]. Treatments were applied for 15 days to three containers per group of unprimed and primed plants (Supplementary Figure S9). The full-strength nutrient solution was composed of (in mM): 1.5 MgSO_4_, 1.6 KH_2_PO_4_, 0.6 K_2_HPO_4_, 3 KNO_3_, 3.5 Ca (NO_3_)_2_, 2 NH_4_NO_3_, MnSO_4_ (0.5), CuSO_4_ (0.04), ZnSO_4_ (0.05), H_3_BO_3_ (0.5), Mo_7_O_24_ (0.02). Fe was supplied as Fe (III)–EDTA.

### 4.3. Fresh Biomass Determination

At the end of the experiment, plants grown under optimal and stressful conditions were harvested and separated into shoots and roots. Roots were carefully rinsed several times with distilled water, paper dried, and then both organs were immediately weighed for the determination of the fresh weight (FW) using a precision balance (Mettler type AE100 at 1/100 of mg). The root-to-shoot ratio (R/S) was subsequently calculated from the corresponding fresh biomass values.

### 4.4. Phytohormone Extraction and Analysis

The main classes of plant hormones, cytokinins (trans-zeatin, tZ, trans-zeatin riboside, ZR, and isopentenyl adenine, iP), gibberellins (GA_4_), indole-3-acetic acid (IAA), abscisic acid (ABA), salicylic acid (SA), jasmonic acid (JA), and the ethylene precursor 1-aminocyclopropane-1-carboxylic acid (ACC) were analyzed following the protocol described previously in [5,56]. Briefly, 0.1 g of fresh shoot or root tissue was homogenized in liquid nitrogen and dropped in 0.5 mL of cold (−20 °C) extraction mixture of methanol/water (80/20, *v*/*v*) for 30 min at 4 °C. Solids were separated by centrifugation (20,000× *g*, 15 min) and re-extracted for 30 min at 4 °C in an additional 0.5 mL of the same extraction solution. Pooled supernatants were passed through Sep-Pak Plus C18 cartridges (SepPak Plus, Waters, Milford, MA, USA) to remove interfering lipids and part of the plant pigments and evaporated at 40 °C under vacuum either to near dryness or until organic solvent was removed. The residue was dissolved in 1 mL of methanol/water (20/80, *v*/*v*) solution using an ultrasonic bath. The dissolved samples were filtered through 13mm diameter Millex filters with 0.22 μm pore size nylon membrane (Millipore, Bedford, MA, USA). Ten μL of filtrated extract was injected into a U-HPLC-MS system consisting of an Accela Series U-HPLC (ThermoFisher Scientific, Waltham, MA, USA) coupled to an Exactive mass spectrometer (ThermoFisher Scientific, Waltham, MA, USA) using a heated electrospray ionization (HESI) interface. Mass spectra were obtained using the Xcalibur software version 2.2 (ThermoFisher Scientific, Waltham, MA, USA). For quantification of the plant hormones, calibration curves were constructed for each analyzed component (0, 1, 10, 50, and 100 μg. L^−1^). Recovery percentages ranged between 92 and 95%.

### 4.5. Statistical Analysis

Data were subjected to a 2-way analysis of variance (ANOVA) to test the main effect of salinity and priming treatment and their interaction. Correlation analysis determined relationships between plant growth and hormonal variables, and for the PCA plots, the varimax method was used. Heatmaps were generated using GraphPad Prism 9.0 (GraphPad Software, San Diego, CA, USA) to visualize relative changes in hormone concentrations and correlation patterns across treatments and species. All analysis were performed using IBM SPSS Statistics 23 (SPSS Inc., Chicago, IL, USA) software. Data normality and homogeneity of variances were verified using Shapiro–Wilk and Levene’s tests, respectively. When significant effects were detected, means were compared using Tukey’s post hoc test at a significance level of *p* ≤ 0.05.

## 5. Conclusions

This study demonstrates that salinity imposes a pronounced inhibitory effect on the growth of barley, with domestic *Hordeum vulgare* being less tolerant than the wild *Hordeum maritimum*, which relies on constitutive tolerance mechanisms characterized by higher levels of growth-promoting hormones (IAA, CKs, GAs) and a more stable ABA regulation. Seed priming with salicylic acid and, to a lesser extent, calcium chloride effectively mitigated the adverse effects of salinity, particularly in *Hv*, by enhancing biomass accumulation, modulating hormonal profiles, and supporting root development. Hormonal analyses revealed species-specific responses under salinity: *Hv* exhibited stress-induced ABA accumulation and reduced JA and Cks (in roots) which were associated with growth inhibition, whereas *Hm* reduced ABA and elevated SA, IAA, JA, CKs and GAs, supporting its higher biomass under salinity. In *Hv*, priming induced a coordinated hormonal reprogramming that rebalanced growth- and stress-related pathways, supporting growth recovery and improved root allocation under salinity, whereas *Hm* depended primarily on constitutive mechanisms with limited responsiveness to priming. This pattern supports the notion that salt-sensitive species such as *Hv* are more responsive to external attenuators like seed priming, whereas inherently tolerant species such as *Hm* derive limited additional benefit due to their constitutive adaptive mechanisms. From a broader perspective, future research should explore (i) the molecular mechanisms underlying priming-mediated hormonal cross-talk, (ii) the long-term impact of priming on reproductive development and yield under saline conditions, and (iii) the applicability of priming strategies across other cereals and halophytes. Additionally, combining priming with other agronomic practices, such as nutrient management or beneficial microbial inoculation, may further optimize crop performance under challenging environments. Such integrative approaches are crucial for sustaining productivity in salt-affected soils and supporting food security in areas prone to salinization.

## Data Availability

Data will be made available on request.

## Supplementary Materials

The following supporting information can be downloaded at: https://www.mdpi.com/article/10.3390/plants15010064/s1, Figure S1: Representative phenotypes of *H. vulgare* and *H. maritimum* under control and saline conditions, with and without seed priming. Figure S2: PCA of total fresh weight (TFW) across four conditions (optimal and saline; unprimed and primed) for both species. Figure S3: Endogenous salicylic acid (SA) content in leaves and roots of *H. vulgare* and *H. maritimum* under control and saline conditions. Figure S4: Endogenous 1-aminocyclopropane-1-carboxylic acid (ACC) content in leaves and roots of *H. vulgare* and *H. maritimum* under control and saline conditions. Figure S5: Correlation matrix between growth parameters and hormonal profiles across treatments and species. Figure S6: PCA biplots of growth and hormonal variables in *Hordeum vulgare* under salinity for unprimed and primed plants. Figure S7: PCA biplots of growth and hormonal variables in *H. maritimum* under salinity for unprimed and primed plants. Figure S8: Conceptual model summarizing growth and hormonal responses in *Hordeum vulgare* and *H. maritimum* under salinity and after priming. Figure S9: Schematic representation of the experimental design.

## Abbreviations

The following abbreviations are used in this manuscript:

*Hv*	*Hordeum vulgare*
*Hm*	*Hordeum maritimum*
ABA	abscisic acid
IAA	indole-3-acetic acid
JA	jasmonic acid
tZ	trans-zeatin
ZR	zeatin riboside
iP	isopentenyl adenine
GA_4_	gibberellin A_4_
ACC	1-aminocyclopropane-1-carboxylic acid
TFW	total fresh weight
SFW	shoot fresh weight
RFW	root fresh weight
R/S	root-to-shoot ratio
