# Supplementary material for: Growth and Hormonal Responses to Salicylic Acid and Calcium Chloride Seed Priming in Domestic and Wild Salt-Tolerant Barley Species Under Saline Conditions
Source: Plants (Basel). 2025 Dec 25;15(1):64. doi: 10.3390/plants15010064 (PMC12787364; doi:10.3390/plants15010064)
Supplement: Supplementary file 1 [file plants-15-00064-s001.zip › Supplementary material.pdf]

Supplementary material

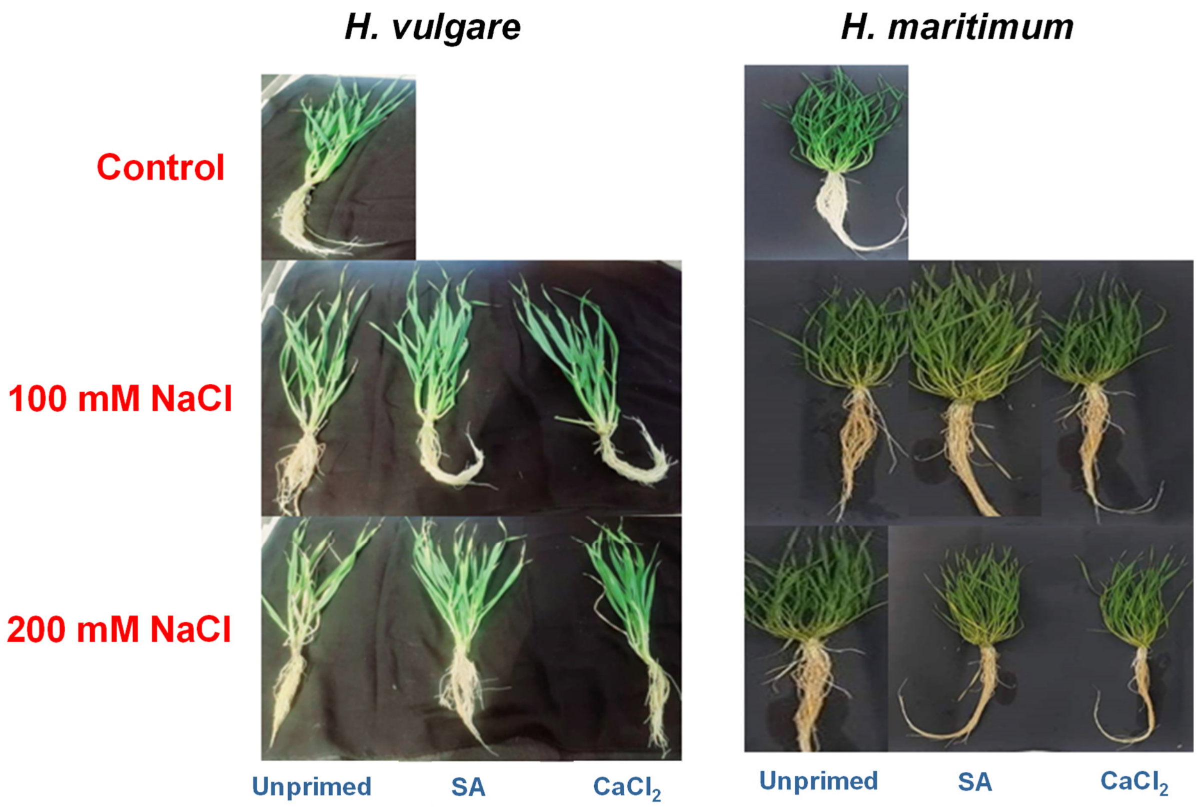

Figure S1. Representative phenotypes of *Hordeum vulgare* and *Hordeum maritimum* plants grown under control, 100 mM, and 200 mM NaCl conditions, with or without seed priming (SA and $CaCl_2$).

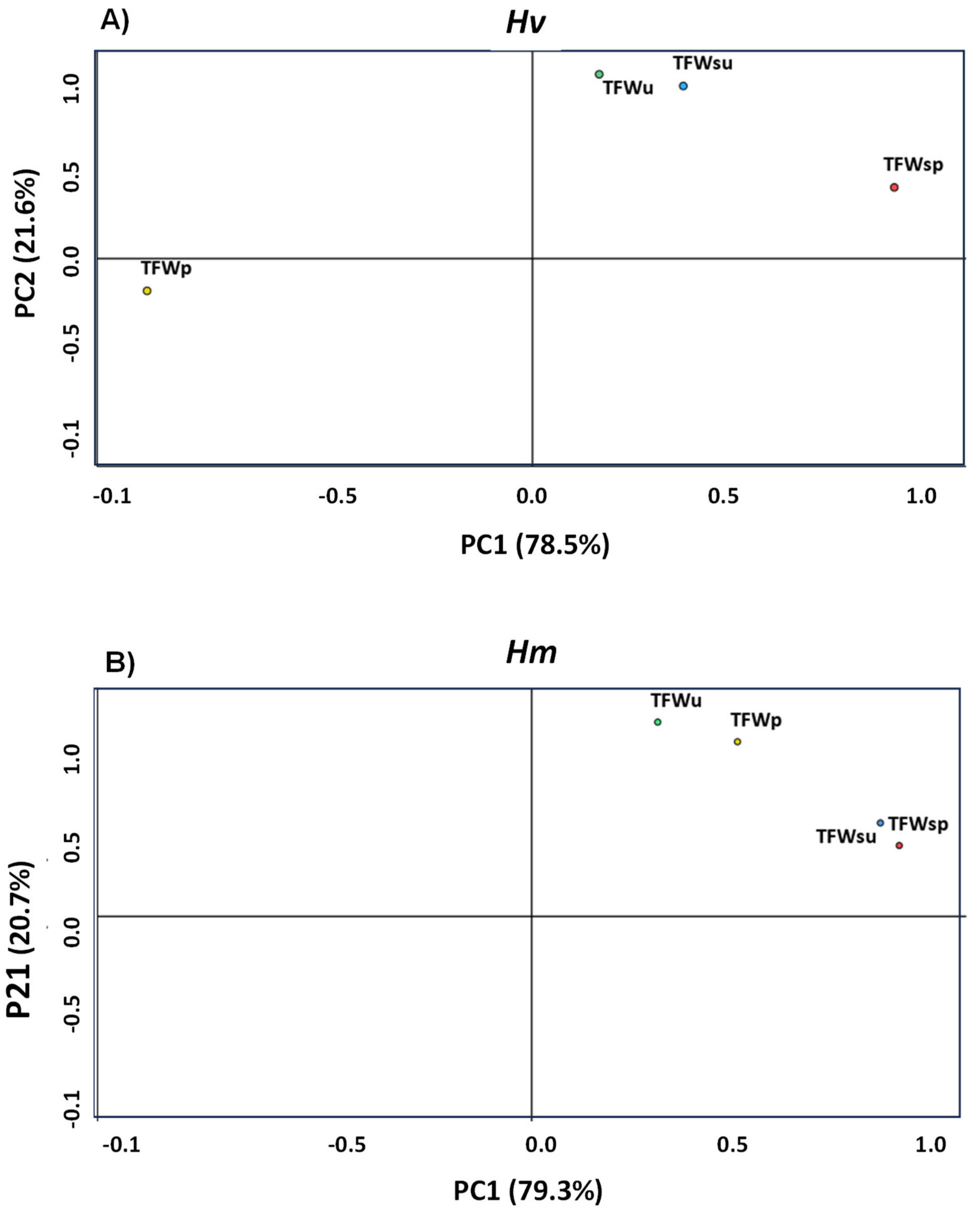

Figure S2. Two axes of a principal component (PC1 and PC2) analysis showing the distribution of total fresh weight (TFW) variable in domestic *Hordeum vulgare* (Hv, A) and wild salt-adapted *Hordeum maritimum*(Hm, B). The analysis includes four conditions: optimal unprimed (TFWu), optimal primed (TFWp), salinityunprimed (TFWsu), and salinityprimed (TFWsp).

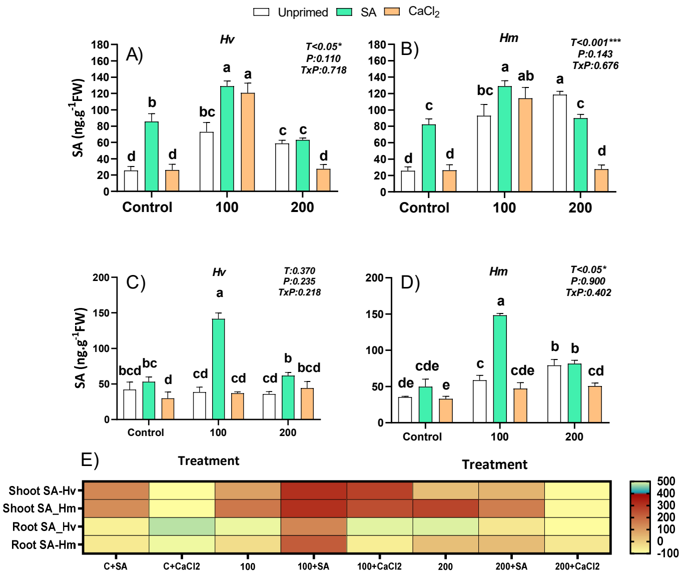

Figure S3. Effect of SA and $CaCl_2$ seed priming on salicilic acid (SA) endogenous content in leaves (A and B) and roots (C and D) in two barley species (*Hv* and *Hm*) exposed to control conditions and two salinity levels (100 and 200mM NaCl) during 15 days. Values are means of three replicates ± standard error. Different letters indicate significant differences among genotypes and treatments according to the Tukey test ($p \leq 0.05$). Results of two-way ANOVA (*p* values reported) for salinity treatment (T), priming treatment (P), and their interaction (T × P) are indicated in the top right of each panel. * and *** indicate statistically significant differences at $p \leq 0.05$ and $p \leq 0.001$, respectively. The heatmap provides a complementary visualization of the data, representing the relative change (%) in hormone levels with respect to the unprimed, non-saline control for each species. This allows a clearer comparison of how salinity and priming treatments modify hormonal profiles in both species (E).

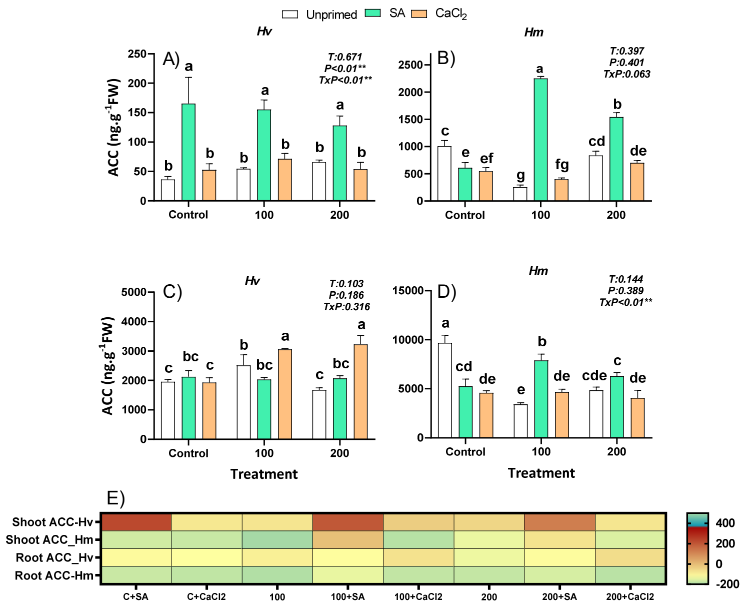

Figure S4. Effect of SA and $CaCl_2$ seed priming on 1-aminocyclopropane-1-carboxylic acid (ACC) endogenous content in leaves (A and B) and roots (C and D) in two barley species (*Hv* and *Hm*) exposed to control conditions and two salinity levels (100 and 200mM NaCl) during 15 days. Values are means of three replicates ± standard error. Different letters indicate significant differences among genotypes and treatments according to the Tukey test ($p \leq 0.05$). Results of two-way ANOVA (*p* values reported) for salinity treatment (T), priming treatment (P), and their interaction (T × P) are indicated in the top right of each panel. ** and *** indicate statistically significant differences at $p \leq 0.01$ and $p \leq 0.001$, respectively. The heatmap provides a complementary visualization of the data, representing the relative change (%) in hormone levels with respect to the unprimed, non-saline control for each species. This allows a clearer comparison of how salinity and priming treatments modify hormonal profiles in both species (E).

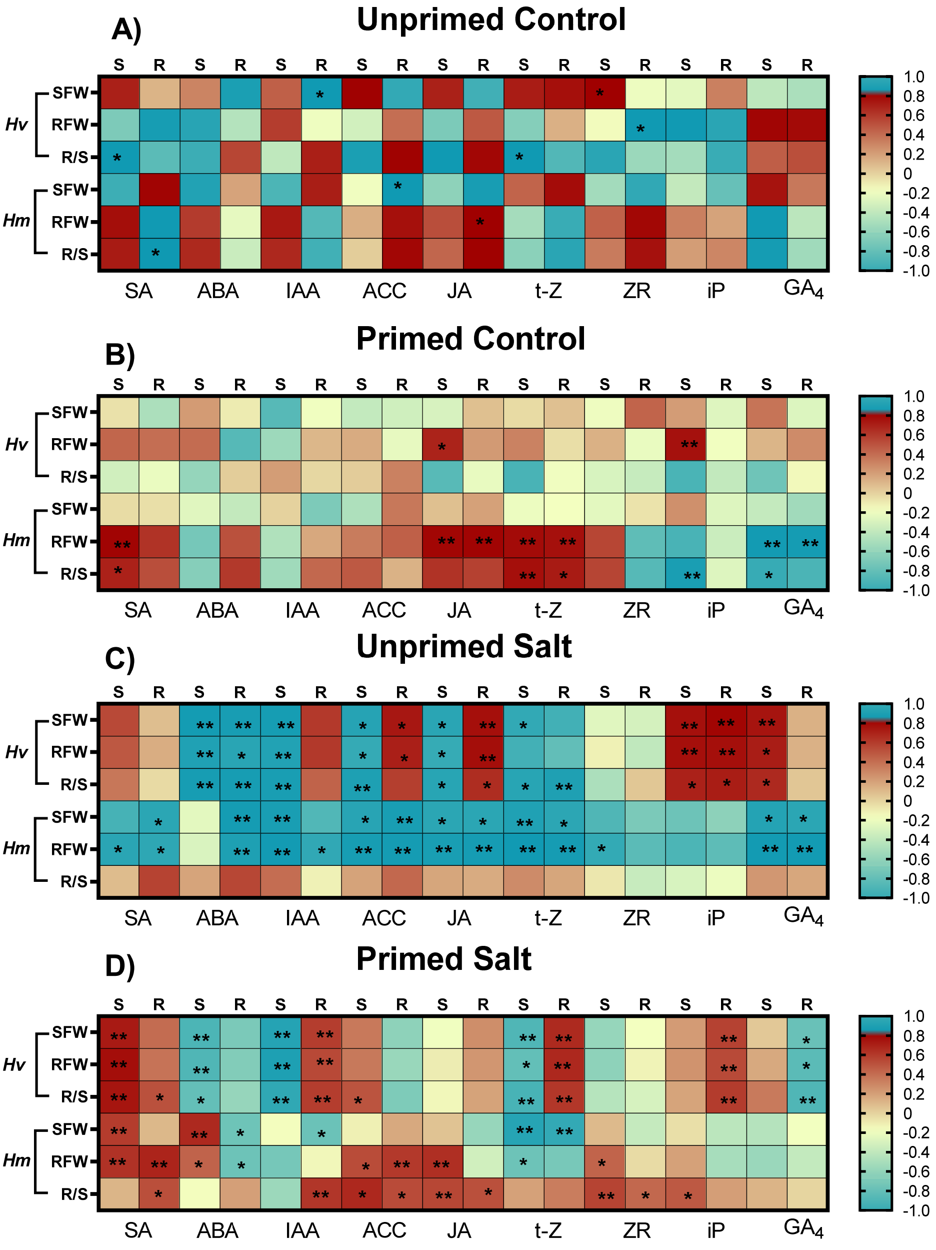

Figure S5. Pearson correlation coefficient heatmaps between growth parameters (SFW, shoot fresh weight; RFW, root fresh weight ; R/S, root to shoot ratio) and hormone concentrations measured in leaves and roots of domestic *Hordeum vulgare* (*Hv*) and wild salt-adapted *Hordeum maritimum* (*Hm*) under four conditions: (A) unprimed control, (B) primed control (SA and $CaCl_2$ combined), (C) unprimed salt-stressed (100 and 200 mM NaCl combined), and (D) primed salt-stressed (SA and $CaCl_2$ combined under 100 and 200 mM NaCl). The color scale represents correlation values from positive (brown) to zero (yellow) to negative (blue). Asterisks indicate statistically significant correlations: $p \leq 0.05$ (*), $p \leq 0.01$ (**), and $p \leq 0.001$ (***).

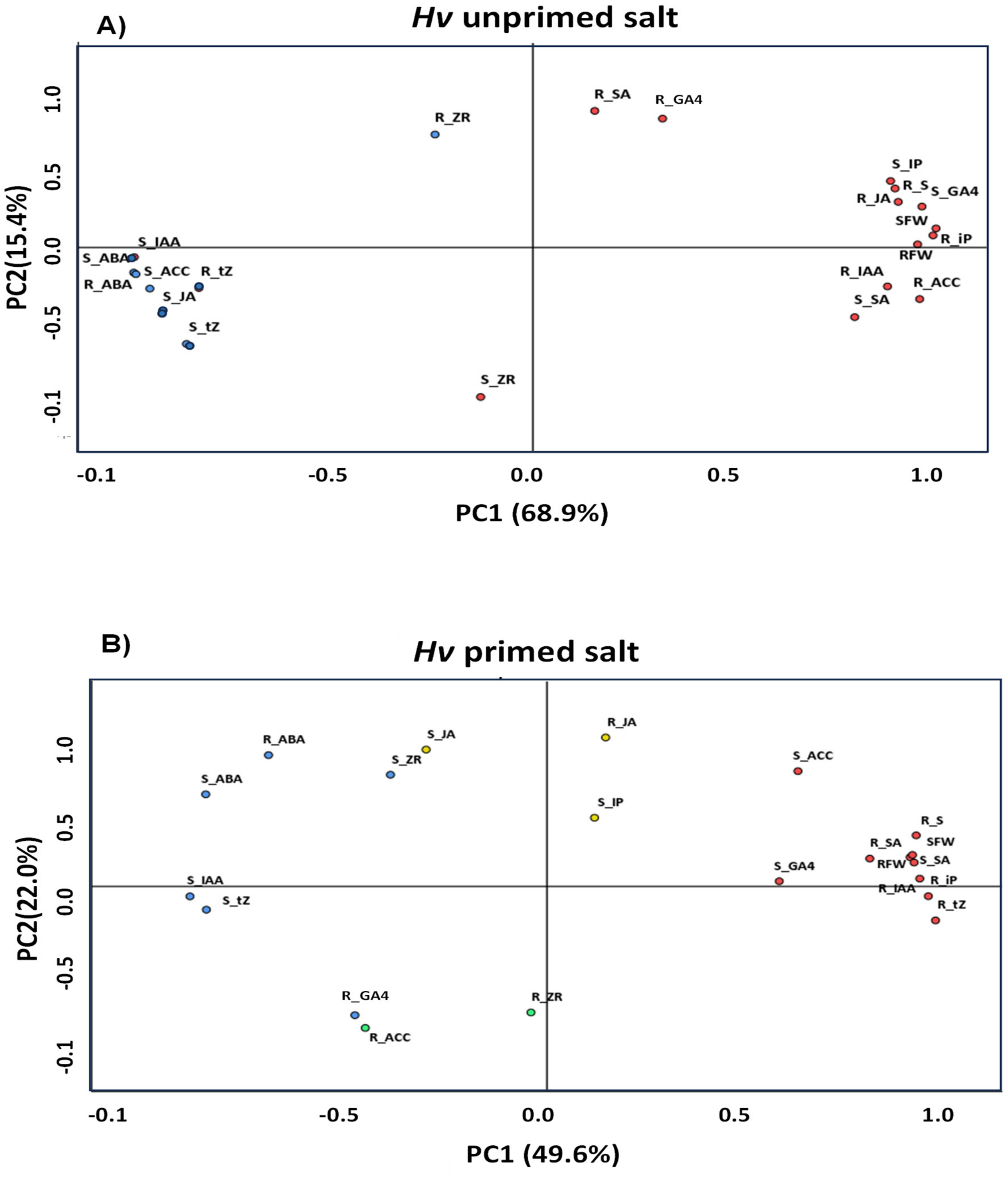

Figure S6. Principal component analysis (PCA) biplots showing the distribution of all measured variables (indicated by abbreviations) along the first two principal components (PC1 and PC2) in *Hordeum vulgare* (*Hv*) under salinity conditions: (A) unprimed and (B) primed. Key variables include: shoot fresh weight (SFW), root fresh weight (RFW), root-to-shoot ratio (R_S), and hormonal compounds such as ACC (1-aminocyclopropane-1-carboxylic acid), ABA (abscisic acid), SA (salicylic acid), JA (jasmonic acid), tZ (trans-zeatin), ZR (zeatin riboside), and GA4 (gibberellin GA4) measured in shoot (S-) and root (R-).The red, blue, yellow, and green circles indicate variables grouped into clusters 1, 2, 3, and 4, respectively, defined at the 80% confidence level.

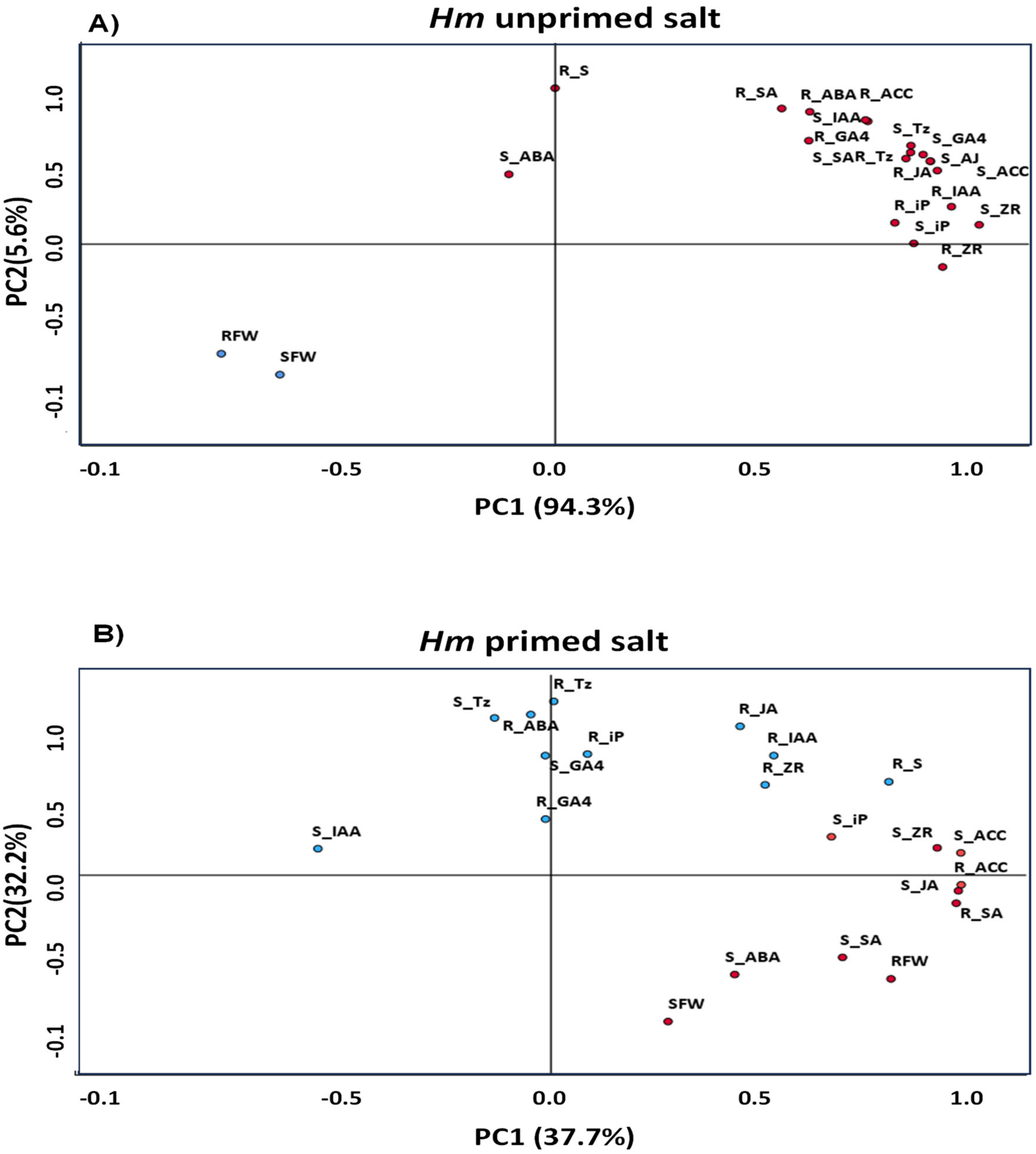

Figure S7. Principal component analysis (PCA) biplots showing the distribution of all measured variables (indicated by abbreviations) along the first two principal components (PC1 and PC2) in Hordeum maritimum (Hm) under salinity conditions: (A) unprimed and (B) primed. Key variables include: shoot fresh weight (SFW), root fresh weight (RFW), root-to-shoot ratio (R_S), and hormonal compounds such as ACC (1-aminocyclopropane-1-carboxylic acid), ABA (abscisic acid), SA (salicylic acid), JA (jasmonic acid), tZ (trans-zeatin), ZR (zeatin riboside), and GA4 (gibberellin GA4) measured in shoot (S-) and root (R-).The red, blue, yellow, and green circles indicate variables grouped into clusters 1, 2, 3, and 4, respectively, defined at the 80% confidence level.

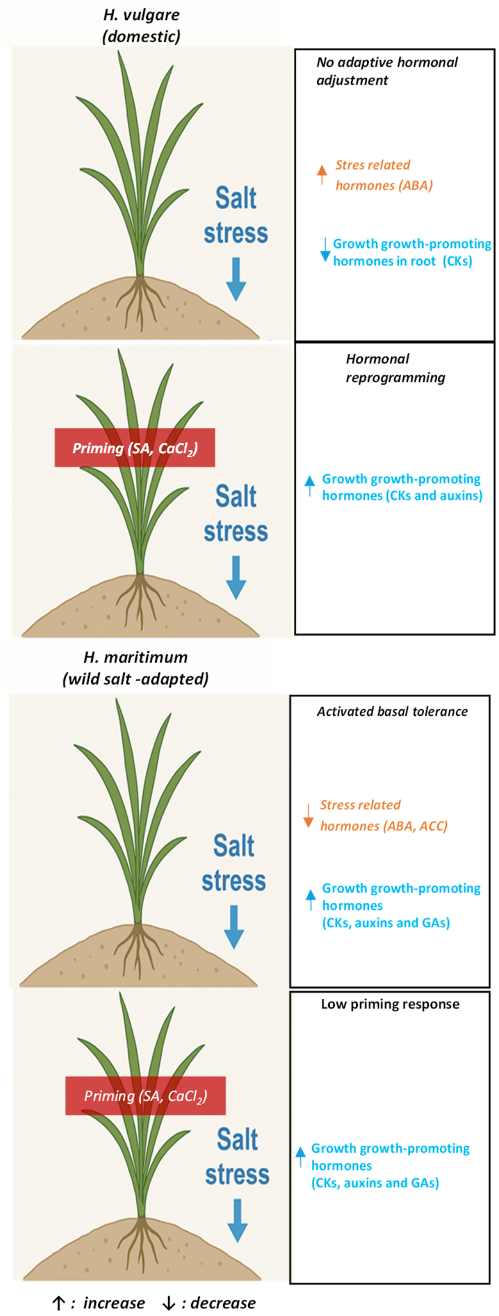

Figure S8. Conceptual model summarizing growth and hormonal responses in Hordeum vulgare (domestic, upper pannel) and H. maritimum (wild, salt-adapted, lower pannel) under salinity and after priming. In Hordeum vulgare, salinity alters hormonal balance—inducing abscisic acid (ABA) accumulation and reducing growth-promoting cytokinins (tZ, iP, and ZR), particularly in roots—which leads to a marked reduction in biomass. Seed priming reprograms hormonal responses, lowering ABA accumulation while enhancing IAA, tZ, and iP accumulation, especially in roots, thereby mitigating salt-induced growth inhibition. In Hordeum maritimum, salinity activates basal tolerance mechanisms characterized by a coordinated hormonal adjustment—reduction of stress-related hormones (ABA and ACC) and stimulation of growth-promoting hormones (IAA, CKs, and GA_4). This adaptive hormonal balance supports continued growth and physiological stability under saline conditions. Arrows indicate hormone changes : ↑ = increase, ↓ = decrease relative to control (unprimed salt) or relative to salinity (primed plants). Stress-related hormones are shown in orange and growth-promoting in blue.

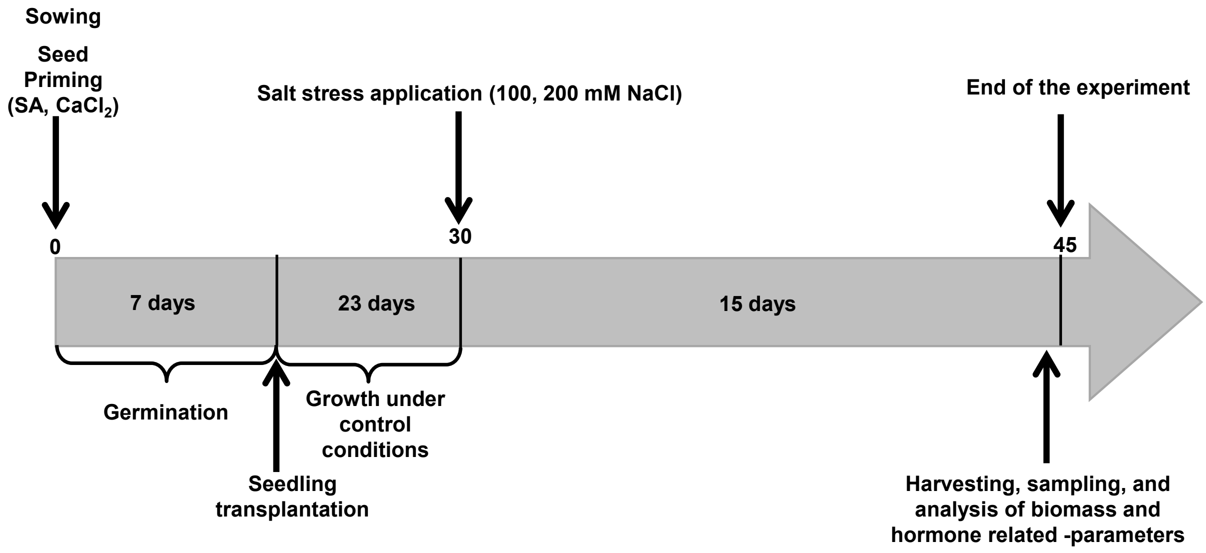

Figure S9. Schematic representation of the experimental design. The diagram illustrates the workflow from seed sterilization and priming treatments (unprimed, salicylic acid—SA, and calcium chloride—$CaCl_2$) through germination and plant growth under control and saline conditions (100 and 200 mM NaCl). It also indicates the sampling stages for growth and hormonal analyses in leaves and roots after 15 days of salt treatment.
