# Supplementary figures and images for: Growth and Hormonal Responses to Salicylic Acid and Calcium Chloride Seed Priming in Domestic and Wild Salt-Tolerant Barley Species Under Saline Conditions
Source: Plants (Basel). 2025 Dec 25;15(1):64. doi: 10.3390/plants15010064 (PMC12787364; doi:10.3390/plants15010064)

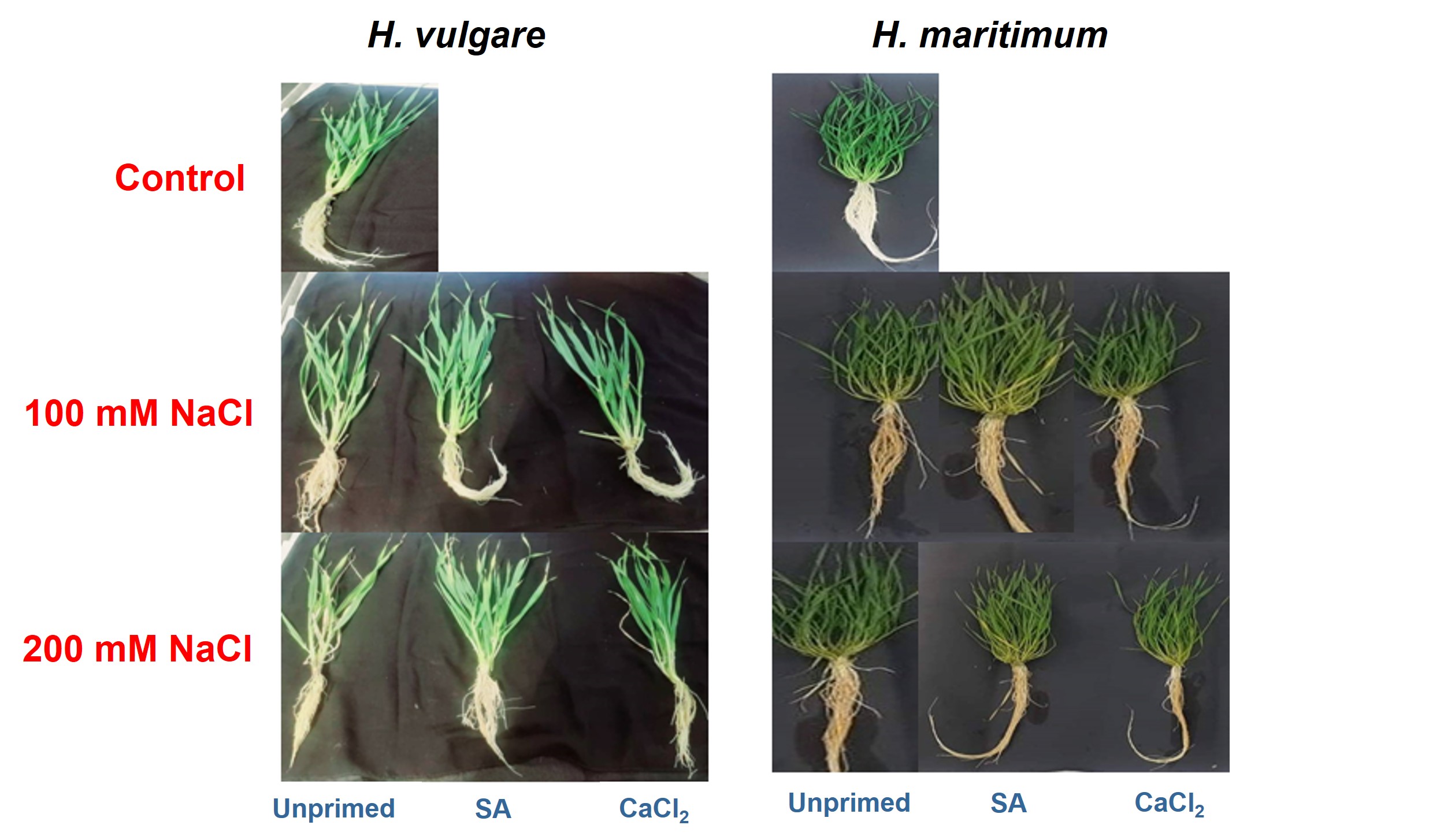

Supplement: Supplementary file 1 [file plants-15-00064-s001.zip › Figure S1 revised.jpg]

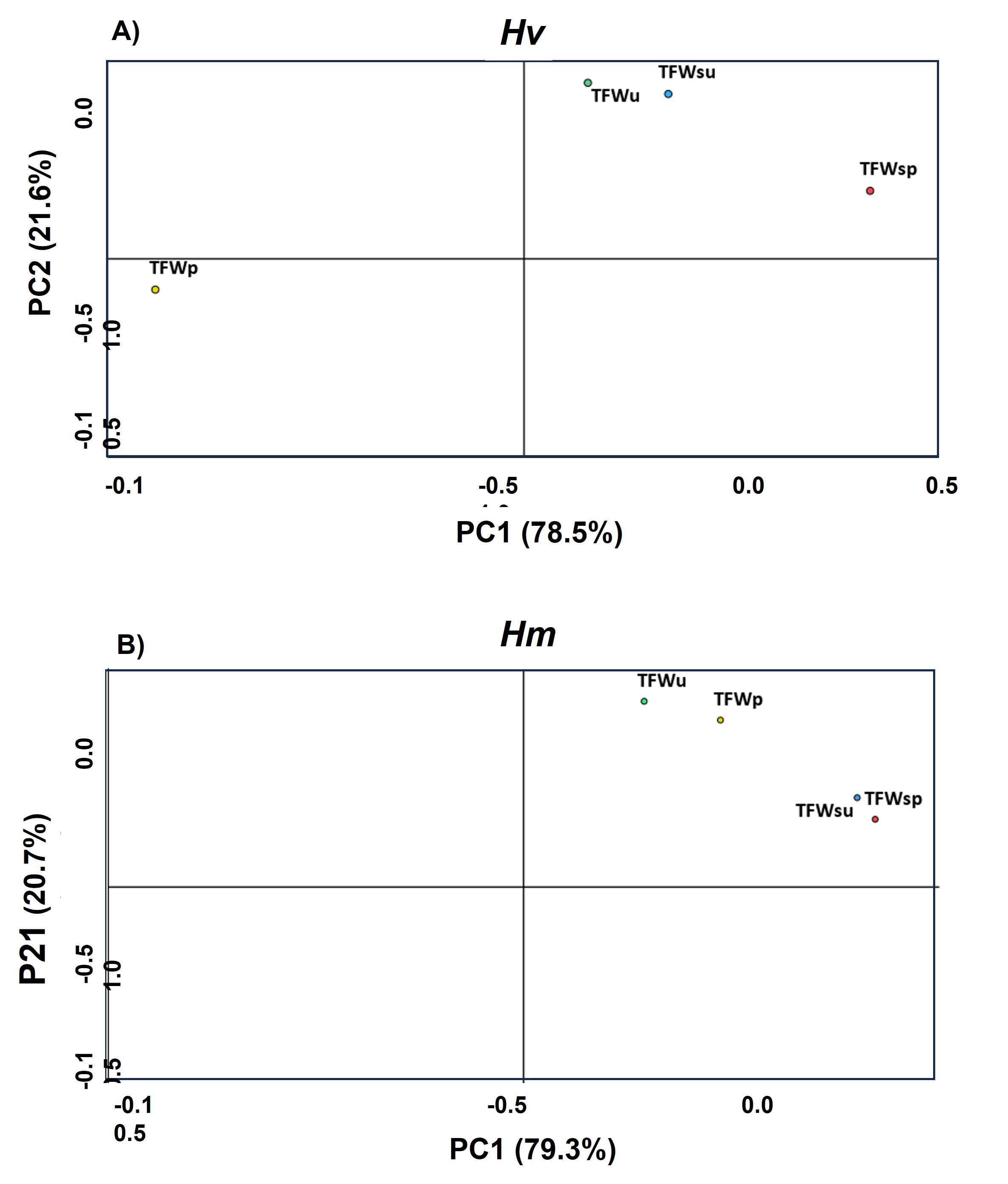

Supplement: Supplementary file 1 [file plants-15-00064-s001.zip › Figure S2 revised.jpg]

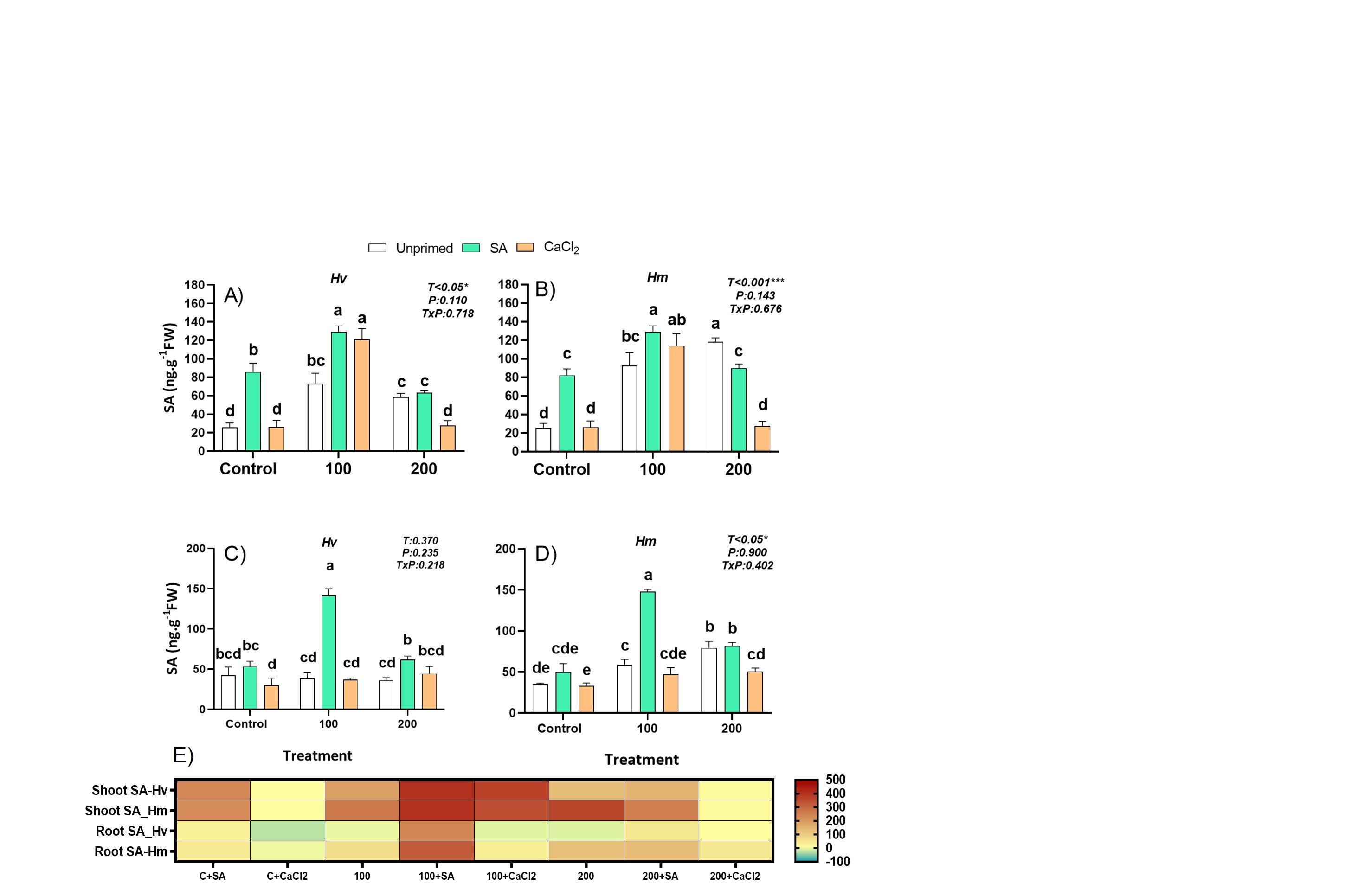

Supplement: Supplementary file 1 [file plants-15-00064-s001.zip › Figure S3 revised.jpg]

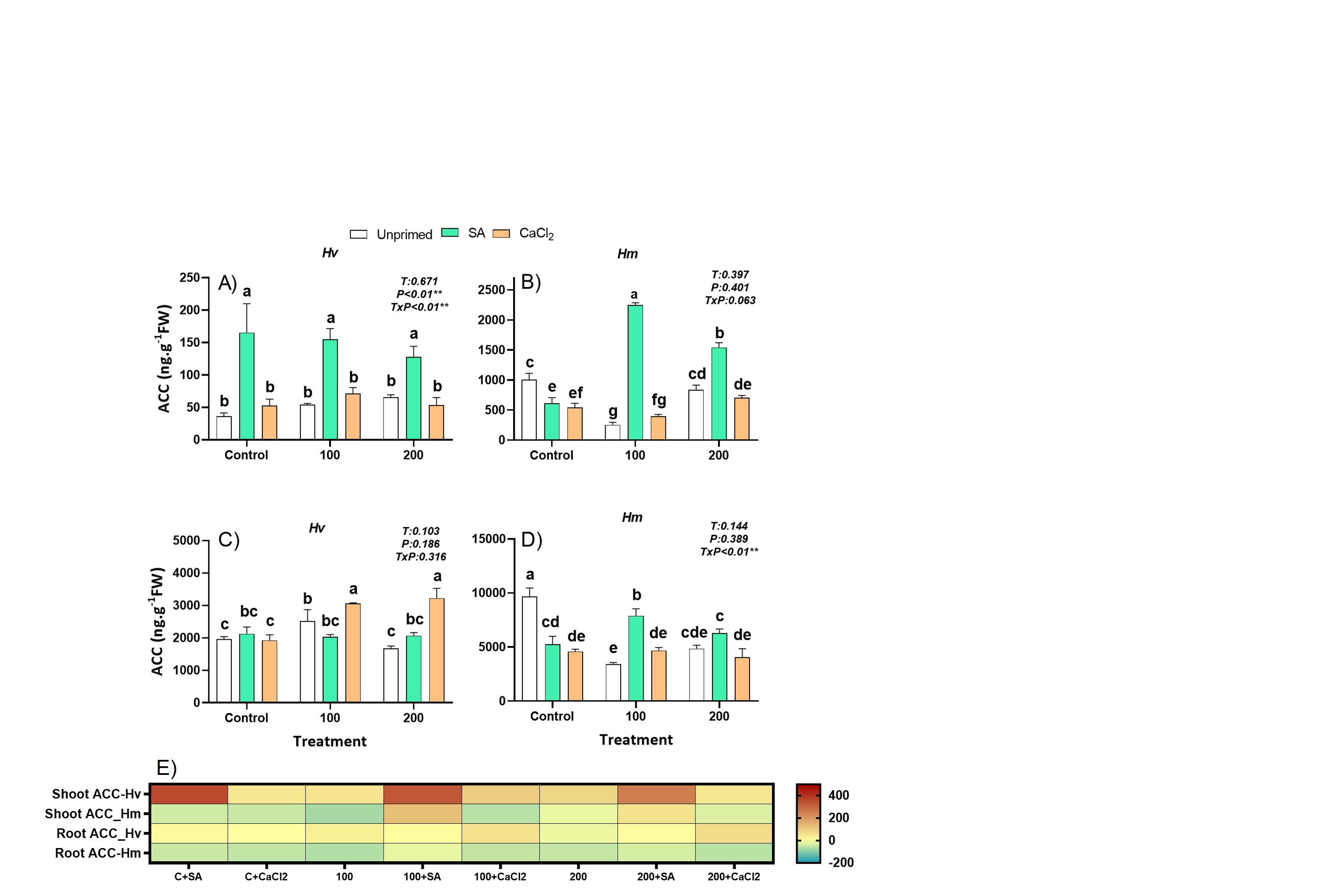

Supplement: Supplementary file 1 [file plants-15-00064-s001.zip › Figure S4 revised.jpg]

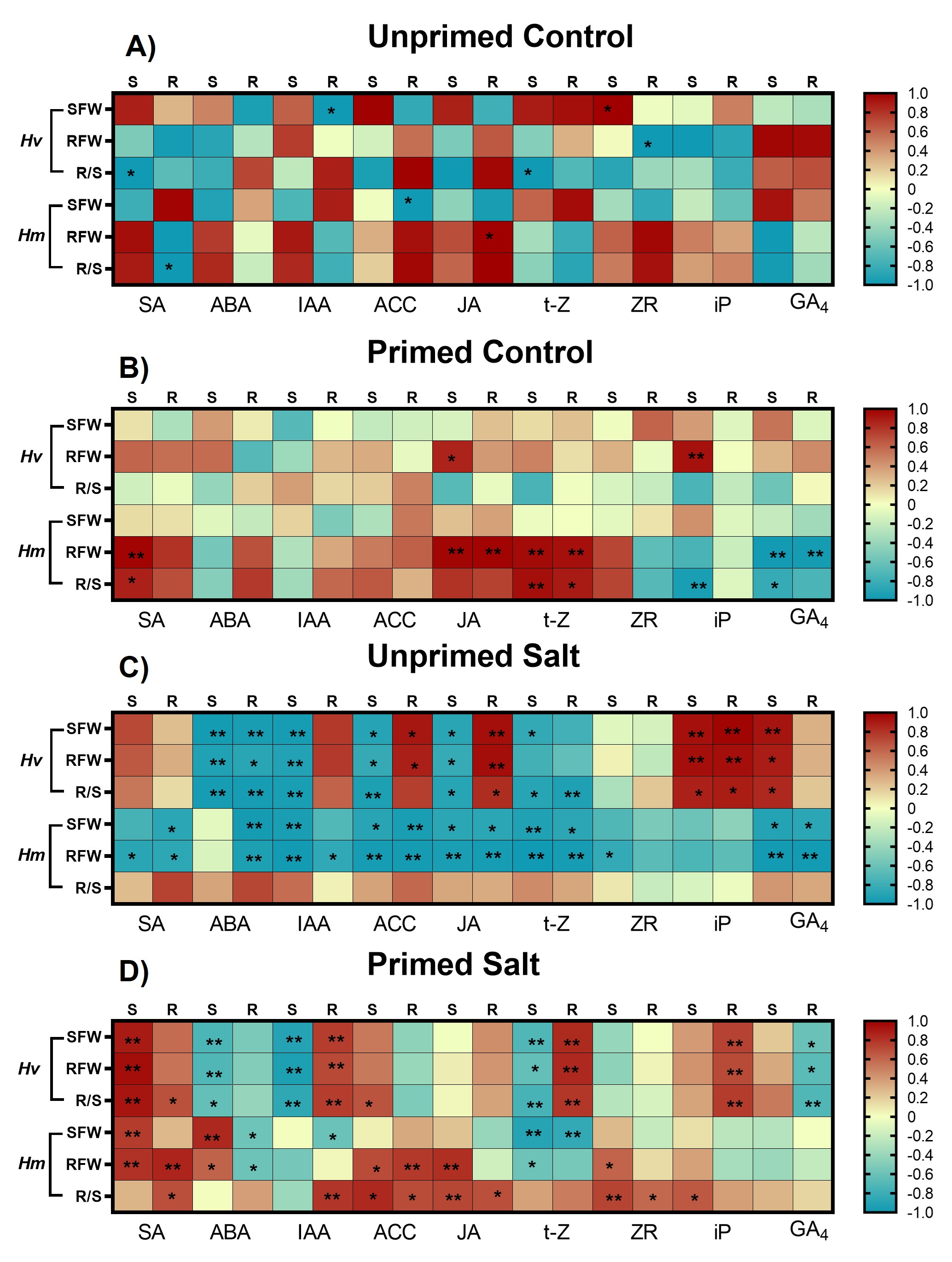

Supplement: Supplementary file 1 [file plants-15-00064-s001.zip › Figure S5 revised.jpg]

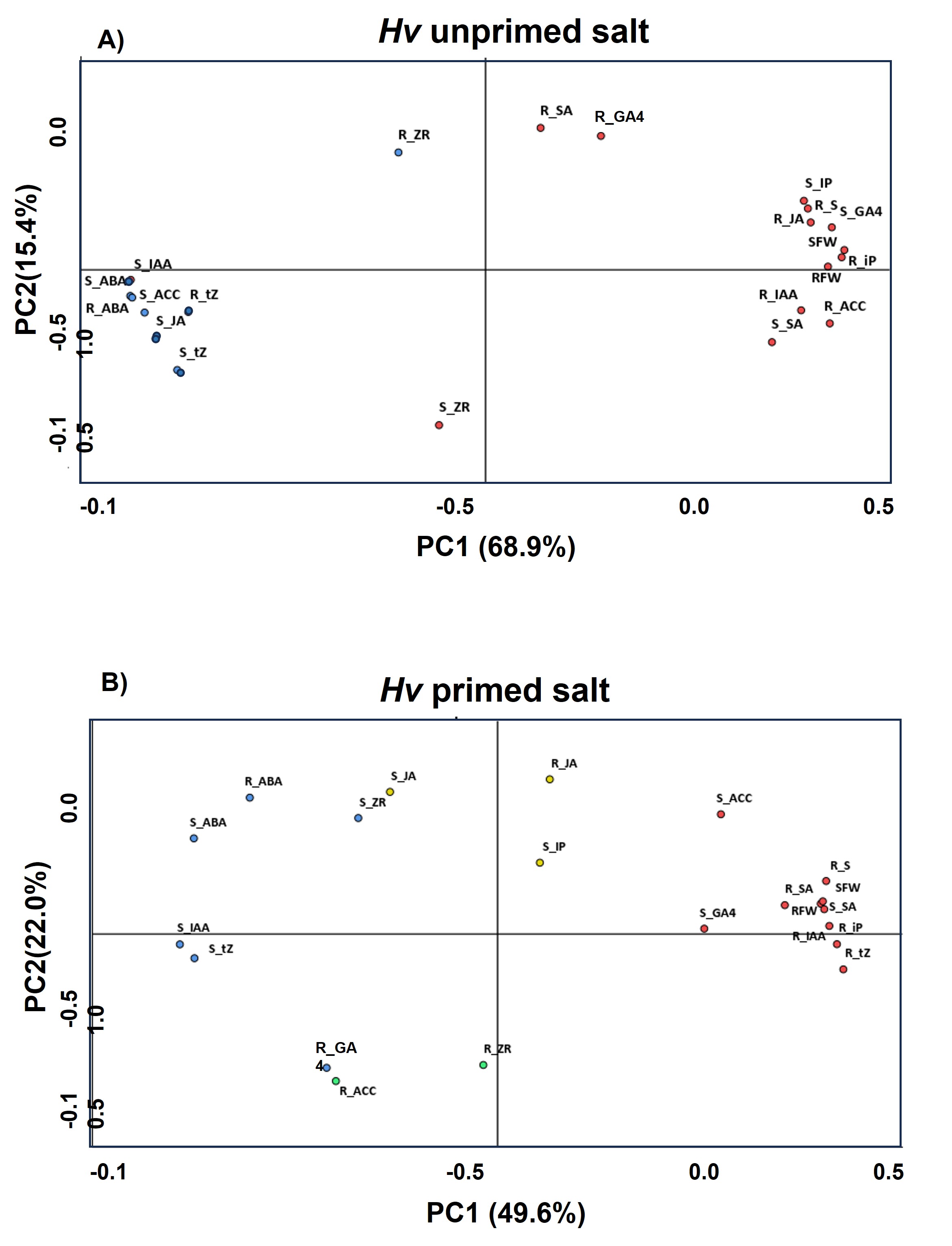

Supplement: Supplementary file 1 [file plants-15-00064-s001.zip › Figure S6 revised.jpg]

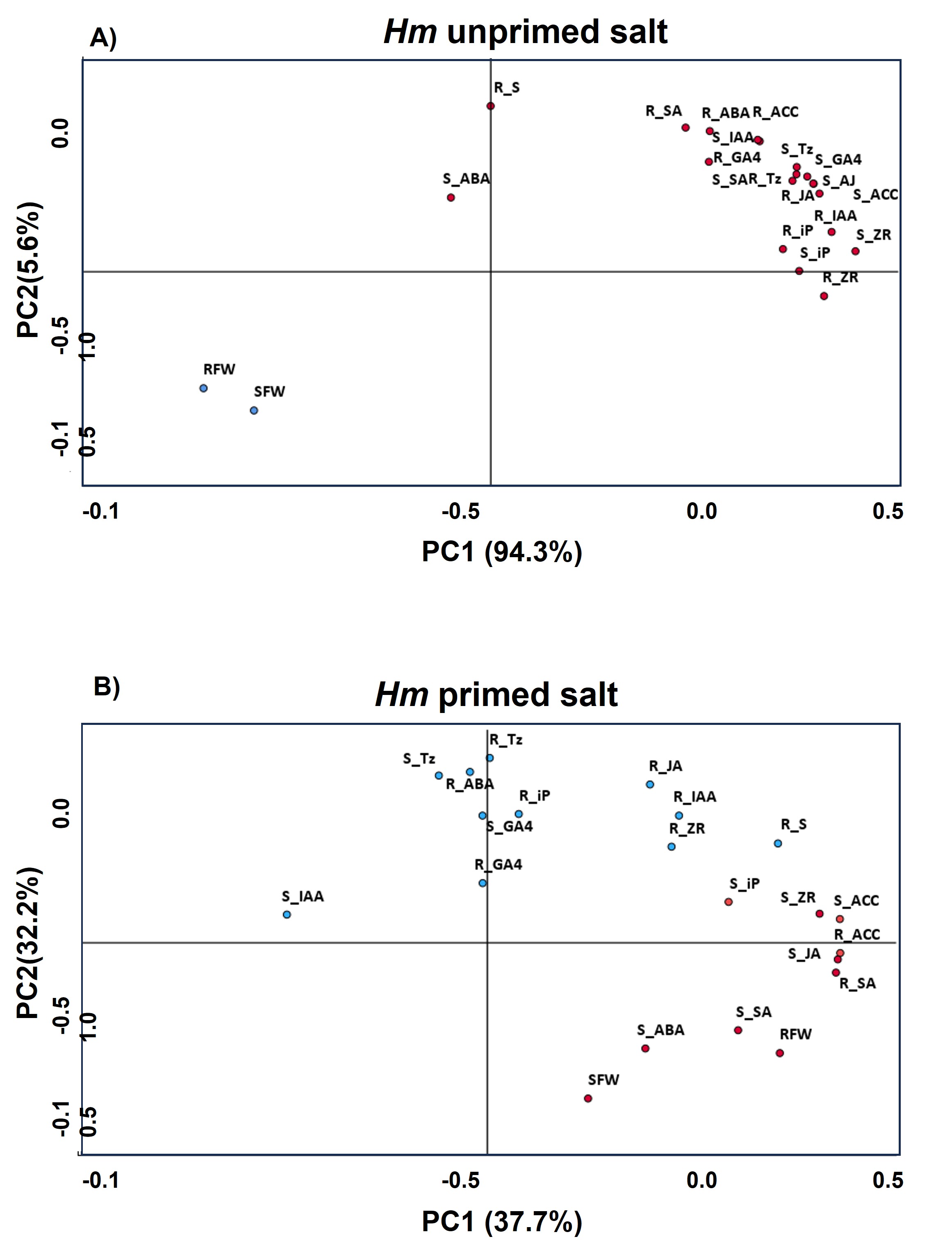

Supplement: Supplementary file 1 [file plants-15-00064-s001.zip › Figure S7 revised.jpg]

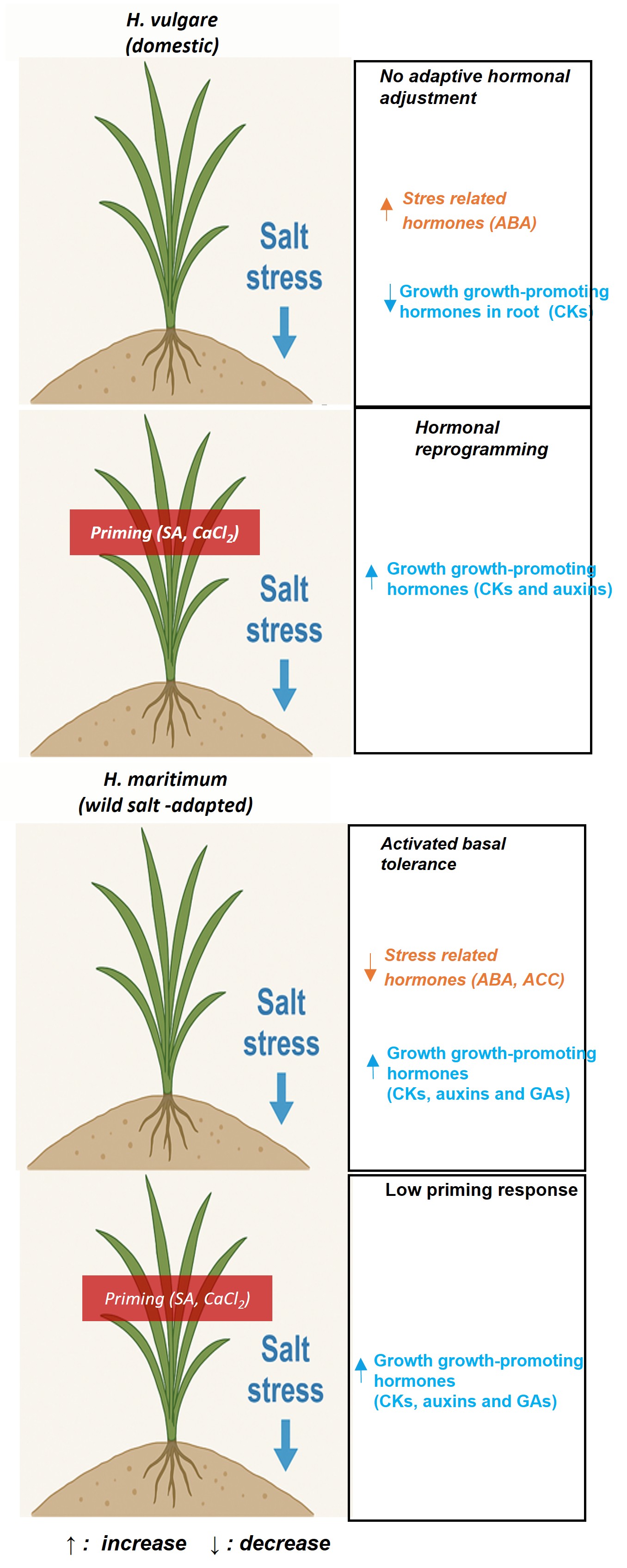

Supplement: Supplementary file 1 [file plants-15-00064-s001.zip › Figure S8 revised.jpg]

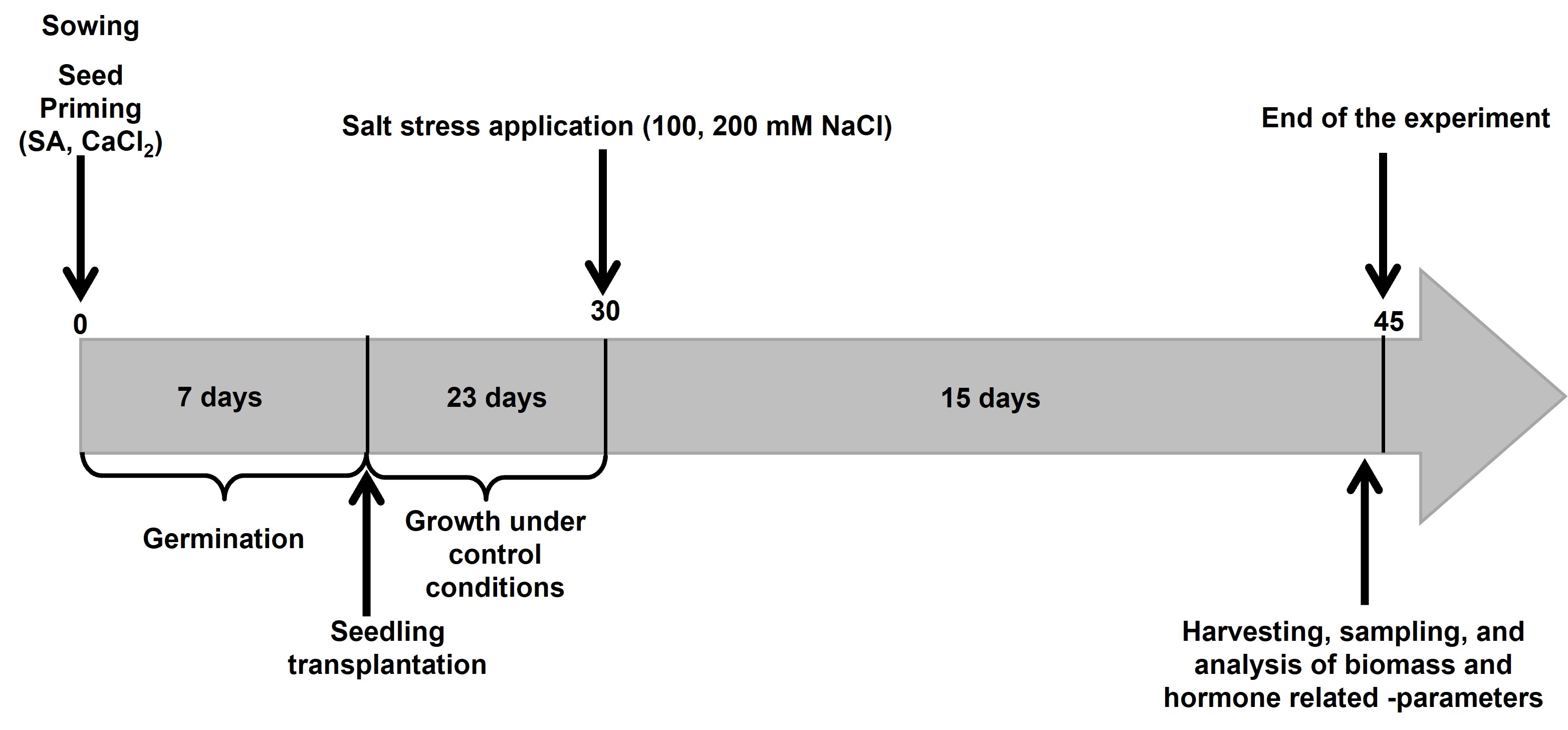

Supplement: Supplementary file 1 [file plants-15-00064-s001.zip › Figure S9 revised.jpg]
